# Enlightening the molecular mechanisms of type 2 diabetes with a novel pathway clustering and pathway subnetwork approach

**DOI:** 10.55730/1300-0152.2620

**Published:** 2022-07-18

**Authors:** Burcu BAKIR-GUNGOR, Miray ÜNLÜ YAZICI, Gökhan GÖY, Mustafa TEMİZ

**Affiliations:** 1 Department of Computer Engineering, Abdullah Gül University, Kayseri, Turkey; 2 Department of Bioengineering, Abdullah Gül University, Kayseri, Turkey

**Keywords:** Genome-wide association study (GWAS), multiple association studies, single nucleotide polymorphism (SNP), subnetwork identification, pathway subnetwork, pathway clustering analysis, type 2 diabetes

## Abstract

Type 2 diabetes mellitus (T2D) constitutes 90% of the diabetes cases, and it is a complex multifactorial disease. In the last decade, genome-wide association studies (GWASs) for T2D successfully pinpointed the genetic variants (typically single nucleotide polymorphisms, SNPs) that associate with disease risk. In order to diminish the burden of multiple testing in GWAS, researchers attempted to evaluate the collective effects of interesting variants. In this regard, pathway-based analyses of GWAS became popular to discover novel multigenic functional associations. Still, to reveal the unaccounted 85 to 90% of T2D variation, which lies hidden in GWAS datasets, new post-GWAS strategies need to be developed. In this respect, here we reanalyze three metaanalysis data of GWAS in T2D, using the methodology that we have developed to identify disease-associated pathways by combining nominally significant evidence of genetic association with the known biochemical pathways, protein-protein interaction (PPI) networks, and the functional information of selected SNPs. In this research effort, to enlighten the molecular mechanisms underlying T2D development and progress, we integrated different in silico approaches that proceed in top-down manner and bottom-up manner, and presented a comprehensive analysis at protein subnetwork, pathway, and pathway subnetwork levels. Using the mutual information based on the shared genes, the identified protein subnetworks and the affected pathways of each dataset were compared. While most of the identified pathways recapitulate the pathophysiology of T2D, our results show that incorporating SNP functional properties, PPI networks into GWAS can dissect leading molecular pathways, and it could offer improvement over traditional enrichment strategies.

## 1. Introduction

Diabetes mellitus (DM) is a group of metabolic disorder that is characterized by high blood sugar levels due to the body’s inability to produce or use insulin. More than 500 million adults struggle with DM, and this number is expected to reach 783 million by 2045 (International Diabetes Federation, 2021) type 1 and type 2 diabetes mellitus (T1D, T2D) are the two main types of diabetes, which contribute to worldwide health care problem by not properly using blood glucose for energy in the body. While T1D is mostly related with pancreatic beta cell damage, T2D is both associated with beta cells’ functionality and insulin resistance (DeFronzo et al., 2015; Zheng et al., 2018); (Piko et al., 2021). Recently, with the help of antidiabetic agents, significant progress has been made in maintaining the glycemic control in T2D patients. Still, the targeted glycated hemoglobin levels could not be maintained for 40% of the adults with diabetes in USA. The decrease in pancreatic beta cell functionality and the increase in the insulin sensitivity of T2D patients over the time, eventually gave rise to the imbalance of glycated hemoglobin (A1C) level and antidiabetic treatment gap (Freeman, 2013). This kind of imbalance and dysfunctionality emerges as a result of the complex interactions among the environmental and genetic risk factors. In this respect, the etiology, driving factors and the genetic predispositions responsible for the increased susceptibility of T2D needed to be well understood in developing new drugs and treatments for this disorder. In this kind of complex diseases, the investigations of different mechanisms of actions may provide benefits for therapeutic approaches. Therefore, postanalysis of high throughput studies conducted at different molecular levels and the elucidation of targeted genes and pathways associated with T2D are crucial.

The widespread introduction of large-scale genetic studies has enabled researchers to investigate the genetic frameworks of complex disorders. During the last decade, genome wide association studies (GWAS) have been widely used to identify the risk factors of complex diseases, to better understand the biological mechanisms of these diseases, and hence to help the discovery of novel therapeutic targets (Claussnitzer et al., 2020). Despite the fact that GWASs has led to a remarkable range of discoveries in human genetics (Visscher et al., 2017), it has some shortcomings. One important shortcoming of GWAS stems from its testing each marker once at a time for association with disease. Since these studies evaluate the significance of the variants individually, they probably miss the SNPs that have low contribution to disease individually, but might be important when interacting collectively (Brubaker et al., 2016; Elmansy and Koyutürk, 2019; López de Maturana et al., 2020). Moreover, in traditional GWASs, the functional effects of significant SNPs, predicted at the splicing, transcriptional, translational, and posttranslational levels are usually neglected. Although GWAS identified more than 140 independent loci influencing the risk of T2D (Scott et al., 2017; Zhao et al., 2017; Mercader and Florez, 2017; Bonàs-Guarch et al., 2018; Mahajan et al., 2018b,a; Xue et al., 2018), most of these loci are driven by common variants and the mechanistic understanding has been achieved only for a couple of these genes (Fuchsberger et al., 2016; Florez et al., 2021). In this respect, post-GWAS strategies need to be developed to enlighten the molecular mechanisms underlying T2D development and progress (Grotz et al., 2017; Meyre, 2017; White et al., 2019).

Several studies indicated that the methods focusing on pathways rather than individual genes can detect significant coordinated changes since these genes act in a synergistic mode in a biological pathway (García-Campos, Espinal-Enríquez and Hernández-Lemus, 2015; Nguyen et al., 2019). Pathway analysis can hypothetically improve power to uncover genetic factors relevant to disease mechanisms, because identifying the accumulation of small genetic effects acting in a common pathway is often easier than mapping the individual genes within the pathway that contribute to disease susceptibility remarkably (Lamparter et al., 2016; Kao et al., 2017; Thrash et al., 2019). The profound discovery that T2D is genetically heterogeneous suggested that the genetic defects might converge on common pathways building up the final similar phenotype (Cirillo et al., 2018; Fernández-Tajes et al., 2019). Besides providing the opportunity to investigate additional therapies that reverse the effects of a particular genetic defect, these findings also may encourage scientists to understand the aberrant networks at genetic, cellular and physiological levels and to devise pharmacological and nonpharmacological intervention strategies.

Inspired by these findings, in this study, we reanalyzed three meta GWAS dataset of T2D, using three different network and pathway-oriented methodologies (top-down approach, bottom-up approach, pathway scoring algorithm) and we presented a new methodology. The first methodology aims to identify disease-associated pathways by combining nominally significant evidence of genetic association with the known biochemical pathways, PPI networks, and the functional information of selected SNPs (Bakir-Gungor, Egemen and Sezerman, 2014). The second methodology finds out dysregulated modules by adding other possible proteins around the known disease protein clusters (Ghiassian et al., 2015). The third methodology calculates pathway scores from SNP-phenotype association summary statistics (Lamparter et al., 2016). Since the pathways are strongly interrelated, in this study we also proposed a new method to identify disease related affected pathway subnetworks and pathway clusters using multiple association studies. In this method, we create a pathway network and then apply subnetwork identification methodologies on the generated pathway network. Our approach is based on both significance level of an affected pathway and its topological relationship with its neighbor pathways. Via testing different subnetwork and pathway-oriented analyses on T2D GWAS metaanalysis datasets, we aimed to enlighten the molecular mechanisms contributing to T2D development.

## 2. Materials and methods

### 2.1. Datasets

#### 2.1.1. 70K for T2D metaanalysis data (T2D1)

Bonàs-Guarch et al. collected T2D genome wide association study (GWAS) data, representing 12,931 cases and 57,196 controls of European ancestry from EGA and dbGaP databases (Bonàs-Guarch et al., 2018). In 70KforT2D metaanalysis data, each dataset was quality controlled and each cohort was imputed to reference panels (1000G and UK10K). Variants which were selected for IMPUTE2 info score ≥ 0.7, MAF ≥ 0.001 and, Hardy-Weinberg equilibrium (HWE) controls p > 1 × 10^−^6, were metaanalyzed. For more details about the followed quality control procedure and association analysis of 70KforT2D dataset, please see, Bonàs-Guarch et al. (2018).

#### 2.1.2. Metaanalysis of DIAGRAM, GERA, UKB GWAS datasets (T2D2)

Xue et al. performed a metaanalysis of GWAS in T2D by gathering DIAGRAM, GERA, UKB GWAS datasets (Xue et al., 2018). A total of 62,892 cases and 596,424 controls of European ancestry were obtained after quality controls and imputed to 1000 Genomes Project. Linkage disequilibrium (LD) score regression analysis was demonstrated. Variants were filtered for GERA and UKB using IMPUTE2 info score ≥ 0.3, MAF ≥ 0.01, HWE controls p > 1 × 10^−^6. Further details about DIAGRAM imputed data in stages 1 and 2, genotyping, quality control and association analysis for each dataset can be found in (Xue et al., 2018).

#### 2.1.3. Type 2 diabetes GWAS metaanalysis dataset (T2D3)

Mahajan et al. collected T2D GWAS datasets from 32 studies including 74,124 cases and 824,006 controls of European population, and aggregated data after initial analyses (Mahajan et al., 2018a). Following quality control checks, the imputation of studies was performed using Haplotype Reference Consortium reference panel, except for deCODE GWAS, where population-specific reference panel was used for imputation. For detailed information, please refer to Mahajan et al. (2018a).

#### 2.1.4. Protein-protein interaction dataset

A human PPI network (interactome data) containing 13,460 proteins and 141,296 protein-protein interactions was derived from (Ghiassian et al., 2015) and used in subnetwork identification steps of this study.

### 2.2. Methods

**Figure 1:**
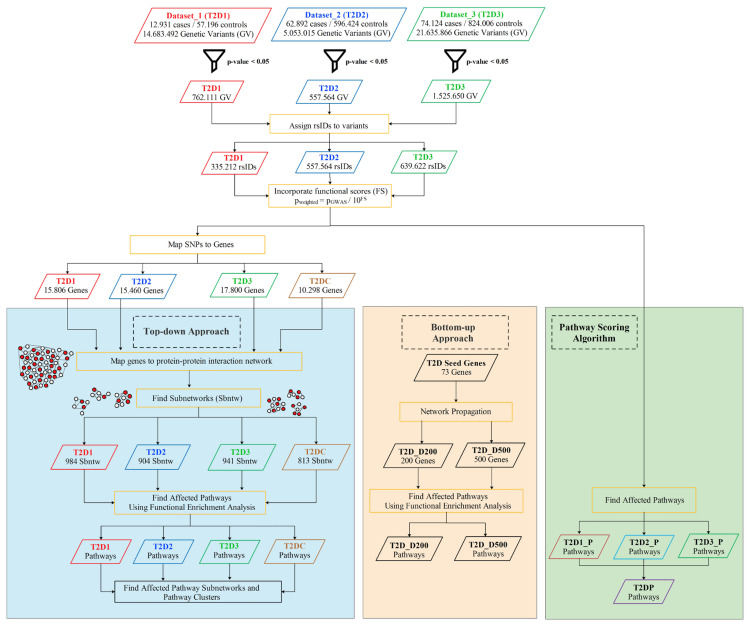
Summary of our pathway and network-oriented approach to enlighten T2D mechanisms using multiple association studies.

To enlighten the molecular mechanisms underlying T2D development and progress, here we integrated different in silico approaches that proceed in top-down manner and bottom-up manner, as summarized in Figure 1. Via combining nominally significant evidence of genetic association with the known biochemical pathways, PPI networks, and the functional information of selected SNPs, our proposed approach identifies disease-associated pathways.

#### 2.2.1. Preprocessing

Association summary statistics for the T2D1, T2D2, and T2D3 datasets were downloaded from each project’s website. This summary statistics data includes i) marker name as chromosome and position, ii) effect allele, iii) noneffect allele, and iv) p-value of association. To be able to assess the collective effect of the variants detected in GWAS with mild effects, all variants were filtered using p < 0.05 cutoff, as suggested in previous studies (Baranzini et al., 2009; Bakir-Gungor and Sezerman, 2011, 2013; Bakir-Gungor et al., 2013, 2015).

#### 2.2.2. Assigning reference SNP cluster IDs (rsIDs) to identified SNPs

While T2D2 dataset provides associated rsIDs of the identified SNPs in the summary statistics data, T2D1 and T2D3 datasets only provide chromosome and position information as marker name of the variants and do not provide associated rsIDs. In this respect, fast and easy variant annotation protocol introduced by (Yang and Wang, 2015) is utilized to assign associated rsIDs to the identified SNPs using hg19 or hg38 reference genomes, depending on the provided genomic coordinates at T2D1, T2D3 datasets.

#### 2.2.3. Assessing the functional impacts of genetic variants

To assess the functional impact of a nonsynonymous change on proteins, numerous computational methods have been developed, as reviewed in (Zeng and Bromberg, 2019). These methods can be classified as follows: i) methods that score mutations on the basis of biological principles, ii) methods that use existing knowledge about the functional effects of mutations in the form a training set for supervised machine learning (Carter et al., 2013). Most of these methods assign a numeric score to the nonsynonymous change, indicating the predicted functional impact of an amino acid substitution. To identify likely functional missense mutations, Douville et al. developed a tool called The Variant Effect Scoring Tool (VEST), that utilizes random forest as a supervised machine learning algorithm (Douville et al., 2016). Douville et al. represented all mutations with a set of 86 quantitative features; and used missense variants from the Human Gene Mutation Database as a positive class and common missense variants detected in the Exome Sequencing Project (ESP) as a negative class, in their training set (Douville et al., 2016). Since VEST scores result in 0.9 sensitivity and 0.9 specificity values, these scores are utilized to assess the functional impacts of genetic variants in our study.

#### 2.2.4. Assigning SNPs to genes

Several post-GWAS studies map disease-associated SNPs to genes based on physical distance (Segrè et al., 2010), LD (Pers et al., 2015), or a combination of both (Wood et al., 2014). In this respect, to aggregate SNP summary statistics into gene scores, several methods have been proposed (Liu et al., 2010; Segrè et al., 2010; Li et al., 2011). Via applying inverse chi-squared quantile transformation on SNP p-values, most of these methods firstly calculate chi-squared values. Secondly, within a window encompassing the gene of interest, some of these methods focus only on the most significant SNP, and assign the maximum of chi-squared as the gene score statistic (Segrè et al., 2010; Lee et al., 2011). Some other methods combine results for all SNPs in the gene region by using the sum of chi-squared statistic (Wang et al., 2011). In order to compute a well-calibrated p-value for the statistic, gene size and LD structure correction is also critical. (Lamparter et al., 2016) rigorously analyzed the effects of using the sum and the maximum of chi-squared statistics, which correspond to the strongest and the average association signals per gene, respectively. (Lamparter et al., 2016) proposed a fast and efficient methodology, Pascal, that calculates gene scores by aggregating SNP p-values from a GWAS metaanalysis (without the need for individual genotypes), while correcting for LD structure. Pascal only requires SNP-phenotype association summary statistics and do not require genotype data. Hence, we utilized this tool in our study to map SNPs into genes.

#### 2.2.5. The identification of dysregulated modules

High throughput experiments enable us to gain better understanding of the functions of the biological molecules in the cell. In addition to the individual activities of these molecules, the molecular interactions are essential to elucidate these molecular mechanisms. In this regard, human PPI networks represent the interactions between human proteins. Since the disease genes tend to physically interact with other disease genes, one strategy for discovering novel disease associated genes is to identify the neighbors of known mediators in the PPI network (Farber and Mesner, 2016; Sonawane et al., 2019). Hence, via analyzing PPI networks, specific sets of proteins (modules) associated with disease phenotype could be detected (Barabási et al., 2011; Ghiassian et al., 2015). This idea is exploited in several post-GWAS analyzes (Bakir-Gungor and Sezerman, 2011; Bakir-gungor and Sezerman, 2013; Bakir-Gungor et al., 2013, 2015; Bakir-Gungor et al., 2014; Chang et al., 2018).

An undirected graph could be defined as G = (V, E), in which the vertex or nodes (V) represent proteins, edges (E) represent the physical interactions among proteins, and graph (G) represent PPI network. A group of proteins in a PPI network that works together to carry out a specific set of functions can be defined as a subnetwork. With the idea of proteins working as a team, disease related protein subnetwork detection has been widely investigated. Active subnetwork search algorithms are originally proposed to identify dysregulated modules in a PPI via utilizing the gene expression values measured in a microarray study (Ideker et al., 2002). The p-values of the genes indicate the significance of expression changes of a gene over certain conditions are mapped to PPI and a search algorithm identifies dysregulated modules. Our group and several others later extended this idea to post-GWAS analyzes, where the SNPs are initially mapped to genes and then the p-values of a gene (genotypic p-values) indicate the significance of a gene in the genetic association study. In this study, to detect dysregulated modules, we use the following two approaches that proceed in top-down and bottom-up manners.

##### 2.2.5.1. Using subnetwork identification algorithms (top-down approach)

The methodology proposed by (Ideker et al., 2002) to identify active modules in PPI networks, became a pioneer study in this field. While this method brings together the nodes that are highly affected by the condition under study, it also gives a chance to the neighbor nodes of these highly affected nodes, even if they are not highly affected. In this method, firstly, a scoring function is defined for each subnetwork and then the problem turned into a search problem of a subnetwork, which maximizes this score. More specifically, to score a subnetwork, the genotypic p-value is converted to a z-score using the equation below, where Φ∧ (−1) indicates inverse normal probability distribution.


$$z_{i}=\Phi^{-1}(1-p_{i})$$

The total z score (*Z_A_*) of the subnetwork A, including k genes is calculated as follows:


$$z_{A}=\frac{1}{\sqrt{k}}\sum_{i\in A}z_{i}.$$

While this score is normalized using the following equation, where *μ* and *σ* indicates mean and standard deviation, respectively; the subnetwork scores are also calibrated by the Monte Carlo method.


$$s_{A}=\frac{\left(z_{A}-\mu_{k}\right)}{\sigma_{k}}$$

Once the subnetwork score is defined, greedy approach, genetic algorithm, and simulated annealing are popular search strategies in active subnetwork identification methodologies. In this study, greedy approach is used during the search steps of the algorithm, and the subnetwork score cutoff is chosen as 3, as suggested in the original paper (Ideker et al., 2002) to select biologically meaningful subnetworks.

##### 2.2.5.2. Using network propagation (bottom-up approach)

Based on the idea that the disease-related proteins do not concentrate in a specific region, studies focus on the estimation of dysregulated modules by using the degree of affected nodes information and edges (protein interaction). (Ghiassian et al., 2015) proposed DIseAse MOdule Detection (DIAMOnD) algorithm that finds out dysregulated modules by adding other possible proteins around the known disease protein clusters. Based on random walking, a defined walker starts from a random seed protein and moves through other nodes along the connections of the network. It is hypothesized that more frequently visited proteins are closer to seed proteins (proteins that are known to be associated with the disease). The probability of a random protein with k interaction having k_s_ interaction with seed proteins is calculated by the hyper-geometric distribution as follows:


$$p(k,k_{s})=\frac{\left(\begin{array}{c} s_{0}\\ k_{s} \end{array}\right)\left(\begin{array}{c} N-s_{0}\\ k-k_{s} \end{array}\right)}{\left(\begin{array}{c} N\\ k \end{array}\right)}.$$

Here, N denotes the number of proteins, s0 denotes the number of seed proteins associated with a particular disease. Whether a protein in the PPI network is randomly interact with the seed protein is calculated by the p-value in equation below. In this way, initiating from seed proteins, other candidate proteins associated with the disease can be identified.


$$p_{value}(k,k_{s})=\sum_{k_{i}=k_{s}}^{k}p(k,k_{i})$$

#### 2.2.6. Functional enrichment

In multifactorial complex disorders, a single factor is unlikely to explain the disease mechanism. Within this scope, functional enrichment analysis focuses on interconnection of terms and functional groups in networks to predict affected pathways for the interested disease. Hypergeometric test and correction methods such as Bonferroni and Benjamini-Hochberg are used for analyses. Hypergeometric p-value determines the significance of gene enrichment above a certain threshold form predefined functional terms. (Total number of genes in organism: f, number of all possible genes in particular pathway: g, number of all differentially expressed genes: d, number of differentially expressed genes in pathway: k)


$$P_{value}=\sum_{k=n}^{\text{min }\;(g,d)}\frac{\left(\begin{array}{c} g\\ k \end{array}\right)\left(\begin{array}{c} f-g\\ d-k \end{array}\right)}{\left(\begin{array}{c} f\\ d \end{array}\right)}$$

In this study, ClueGO (Bindea et al., 2009) is utilized for performing enrichment analysis. Kyoto Encyclopedia of Genes and Genomes (KEGG) biological pathways are used as reference pathways.

For each dataset (T2D1, T2D2, T2D3), firstly the enriched KEGG pathways are listed for each identified subnetwork. For each dataset, a final list of affected pathways is defined by following the methodology developed in our earlier studies (Bakir-Gungor et al., 2012, 2014) and used in (Bakir-Gungor et al., 2013, 2015b; Bakir-Gungor and Sezerman, 2013).

#### 2.2.7. Construction of pathway network

**Figure 2:**
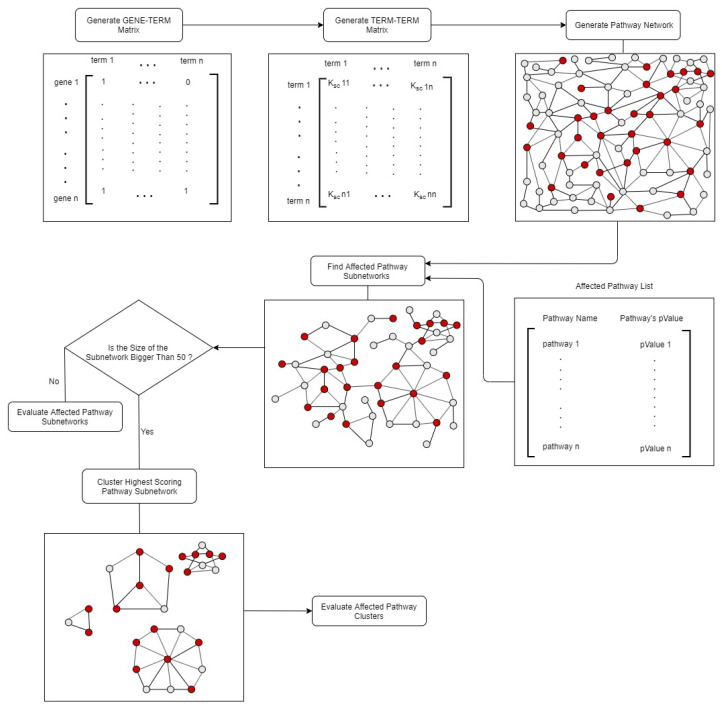
Flowchart of pathway network generation and pathway subnetwork identification.

If two or more biological processes are performed by similar sets of genes, these processes might be somehow related in the biological network. The identification of related pathway terms or biological processes can help biologists to better understand the bigger biological picture. In this regard, we proposed to construct a pathway network and then to detect affected pathway subnetworks. Figure 2 summarizes our steps regarding pathway network generation and pathway subnetwork identification. In order to establish a pathway network, first, the relationships between the genes and 288 KEGG biological pathways need to be analyzed. This relationship is revealed via examining whether the gene of interest is found in a specific pathway or not. For example, if pathway i includes gene j, a value of 1 is assigned to index_i,j_ in the gene-term matrix and if not, a value of 0 is given to this index. Hence, the created gene-term matrix is a binary matrix, as shown in Figure 2. Secondly, the relationships between pathways need to be analyzed. For this purpose, the term-term matrix is formed by using the previously obtained gene-term matrix, as illustrated in Figure 2. Initially suggested by Huang et al. (2007), the Kappa score became a popular metric to determine the relationships between pairs of pathways via calculating the observed cooccurrence and random cooccurrence values (Huang et al., 2009a,b; McHugh, 2012; Brocca et al., 2019; Ulgen et al., 2019; Mlecnik et al., 2019). Since the Kappa score also adjusts the observed cooccurrence with chance cooccurrence, it is a corrected measure of cooccurence. While the higher Kappa values indicate higher cooccurrence and hence stronger agreement; the lower Kappa values indicate weaker agreement. If Kappa score is calculated as 0 for a pathway pair, it indicates that this pathway pair cooccurrence is no better than random chance. The equation expressing the Kappa score for any two pathways A, B is given as follows:


$$\begin{array}{l} G_{A,B}=\frac{{CN}_{1,1}+{CN}_{0,0}}{{CN}_{1,1}+{CN}_{0,0}+{CN}_{0,1}+{CN}_{1,0}}\\ C_{A,B}=\frac{\left({CN}_{0,1}+{CN}_{1,1})*({CN}_{0,1}+{CN}_{1,1})+({CN}_{0,0}+{CN}_{1,0})*({CN}_{0,0}+{CN}_{0,1}\right)}{\left({CN}_{1,1}+{CN}_{0,0}+{CN}_{0,1}+{CN}_{1,0})*({CN}_{1,1}+{CN}_{0,0}+{CN}_{0,1}+{CN}_{1,0}\right)}\\ K_{A,B}=\frac{G_{A,B}-C_{A,B}}{1-C_{A,B}}, \end{array}$$

where G_A,B_ represents the observed cooccurrence, C_A,B_ represents random cooccurrence and K_A,B_ represents the Kappa score between pathways A and B. CN_1,1_, CN_0,0_, CN_1,0_, CN_0,1_ counters are calculated as follows. If the gene of interest is present in both compared pathways, CN_1,1_ counter is increased by 1. Following the same idea, the values of other counters are calculated. Kappa scores, which express the relationships between pairs of pathways, was obtained using observed cooccurrence (G) and random cooccurrence (C) values and stored in term-term matrix. Via applying a threshold on Kappa scores, human KEGG pathway network is created. The pathway network generation steps are implemented in Java.

#### 2.2.8. The identification of affected pathway subnetworks and pathway clusters

To be able to utilize the interrelated structure of the pathways, we proposed to apply subnetwork identification methodologies on the generated pathway networks, hence disease related affected pathway subnetworks could be identified. A classical subnetwork identification algorithm requires the following two information: i) the biological network file, ii) significance of the nodes. In the regular subnetwork identification problem, while (i) refers to a PPI network, (ii) refers to the significance values of the genes, obtained in a microarray experiment. Here, for (i), we used the pathway network that we generated as described in subsection 2.2.7. Regarding (ii), the functional enrichment step, as explained in subsection 2.2.6 outputs affected pathway lists with their p-values, indicating the importance of a pathway for the phenotype under study. Hence, to obtain the affected pathway subnetworks, a similar methodology, as described in subsection 2.2.5.1 is followed. Instead of using a PPI network, in this step, the generated pathway network, as explained in subsection 2.2.7, is used. Instead of using the significance values of the proteins, in this step, the significance values of the pathways, generated in Functional Enrichment Step, subsection 2.2.6, is used. To select biologically meaningful subnetworks among all generated subnetworks, the subnetwork score cutoff is chosen as 3, as suggested in the original paper (Ideker et al., 2002). If the size of the identified subnetwork is bigger than 50, this pathway subnetwork is further subdivided to find disease related pathway clusters. At this step, we used a graph theoretic clustering algorithm, Molecular Complex Detection (MCODE) to discover densely connected pathway clusters in the T2D affected pathway subnetwork (Bader and Hogue, 2003). In order to confine the dense regions in a PPI, MCODE exploits vertex weighting by local neighborhood density and outward traversal from a locally dense seed protein. In our problem setting, while the PPI refers to the generated pathway network, proteins refer to the pathways. The advantage of MCODE over other graph clustering methods is its allowance for the i) fine-tuning of clusters of interest without considering the rest of the network and ii) inspection of cluster interconnectivity, which is relevant for pathway networks (Bader and Hogue, 2003). It uses 4 different parameters to find clusters: cut off value, K-core value, haircut and fluff parameters. The cut off value sets the intensity of the cluster to be estimated. The K-core parameter allows to assign weights to the nodes, which is later used by MCODE to reduce the running time complexity. The haircut parameter, which is a binary parameter, allows the elimination of nodes considered to be topologically irrelevant. The fluff parameter allows someone to set the size of the cluster, which is estimated topologically in the default mode (Bader and Hogue, 2003). In our analyses, the default values of these parameters are used. In the last step, the identified T2D affected pathway subnetworks and pathway clusters are evaluated.

#### 2.2.9. Pathway scoring algorithm (Pascal)

Integration of SNPs across genes and pathways in GWASs has potential to make significant advancement in statistical power and in enlightening relevant biological mechanisms. However, this process is challenging because of the multifunctional roles of genes in several biological processes and the inadequate information about all phenotype – process pairs. In this regard, Pascal is a robust tool to calculate gene and pathway scores from SNP-phenotype association summary statistics (Lamparter et al., 2016). It does not require genotype data. Firstly, they calculate gene scores by aggregating SNP p-values from a GWAS metaanalysis, and also by correcting for LD structure. While computing the gene scores, they compared the effect of using the sum of chi-squared statistics (average association signals per gene) with the effect of using max of chi-squared statistics (strongest association signals per gene) (Lamparter et al., 2016). Secondly, they calculate pathway scores via aggregating the scores of genes that belong to the same pathways by using modified Fisher method (Lamparter et al., 2016).

#### 2.2.10. Comparison of the identified subnetworks and pathways from different datasets using normalized mutual information (NMI)

In order to evaluate the similarities between two different community detection algorithms, (Xuan Vinh et al., 2010) and (Tripathi et al., 2016) proposed to use normalized mutual information. Let U and V be the sets of subnetworks that are identified using different datasets. Let U= {U_1_, …., U_R_} denote the set of R different subnetworks identified using dataset x, and let V= {V_1_, …., V_S_} denote the set of S different subnetworks identified using dataset y. The following contingency table (Table 1) illustrates the numbers of shared genes between pairs of subnetworks. In other words, n_ij_ indicates the number of common genes between subnetworks U_i_ and V_j_. The entropy of communities H(U), H(V) and mutual information I (U, V) are calculated as follows:


$$\begin{array}{l} H(U)=-\sum_{i=1}^{R}\frac{a_{i}}{N}\left(\text{log}\frac{a_{i}}{N}\right)\\ H(V)=-\sum_{i=1}^{S}\frac{b_{i}}{N}\left(\text{log}\frac{b_{i}}{N}\right)\\ I(U,V)=\sum_{i=1}^{R}a\sum_{i=1}^{S}\frac{n_{ij}}{N}\left(\text{log}\frac{n_{ij}/N}{a_{i}b_{j}/N^{2}}\right)\\ {NMI}_{SUM}=\frac{2\times I(U,V)}{H(U)+H(V)}. \end{array}$$

Here, I (U, V) indicate the amount of information shared between U and V communities. NMI_SUM_ is used to compare the clusters in the range of [0,1], where the value 0 refers no similarity between clusters (Vinh et al., 2010). Inspired by these studies, in this research effort firstly we have calculated the numbers of common genes between subnetworks Ui and Vj. Secondly, we have computed the entropy of communities H(U), H(V) and the mutual information I (U, V).

## 3. Results

Based on the idea that the genes and proteins perform cellular functions in a coordinated fashion, understanding the cooperations of proteins in interaction networks may help to identify candidate biomarkers. In this study, we proposed an integrative approach that concurrently analyzes multiple association studies, the functional impacts of these variants, incorporates the interaction partners of susceptibility genes, detects a pathway network of functionally enriched pathways and finally determines the clusterings and subnetworks of affected pathways. The methodology proposed in Figure 1 is applied on three metaanalyses of GWAS data, which are introduced in subsection 2.1. As summarized in Table 2, T2D1, T2D2 and T2D3 datasets include 14,683,492, 5,053,015 and 21,635,866 SNPs, respectively. After the filtration of 3 GWAS datasets using p < 0.05 cutoff, the SNPs with mild effects are collected and the numbers of genetic variants are reduced to 762,111, 557,564 and 1,525,650, for T2D1, T2D2 and T2D3 datasets, respectively. Chromosomal position, reference allele, altered allele information of genetic variants are utilized to assign rsIDs. 335,212 and 639,622 rsIDs are assigned to T2D1 and T2D3 datasets, as explained in subsection 2.2.2 (Reference genome: hg19). 557,564 rsIDs presented as part of T2D2 dataset is used for further analyses. In the next step, functional scores are assigned to each SNP via using VEST (Douville et al., 2016), as explained in subsection 2.2.3. Weighted p-values (p_W_) are calculated for SNPs via combining the genetic association p-values with functional scores (FS) p_w_ = p_GWAS_/10_FS_, as proposed by Saccone et al. (2008). Then, SNPs are mapped to 15,806, 15,460 and 17,200 genes for T2D1, T2D2 and T2D3 datasets, respectively. Combined p-values of 10,298 common genes among three datasets are calculated using Fisher’s combined test (Fisher, 1934), and called as T2D-combined (T2DC) in the rest of this paper. For the detection of dysregulated modules, top-down and bottom-up approaches are followed, as explained in subsection 2.2.5, and as illustrated in Figure 1.

### 3.1. Affected subnetworks that are identified using meta GWAS datasets and top-down approach

In order to identify affected subnetworks, the genes and their significance levels are mapped to PPI network for all datasets. 983, 903, 940 and 813 active protein subnetworks are identified for T2D1, T2D2, T2D3 and T2DC datasets, respectively. We analyzed whether there is any bias among the sizes of the generated subnetworks (in terms of gene numbers) when different T2D GWAS datasets are used. While most of the subnetworks included 175–250 genes in T2D1 and T2D2 datasets, most of the subnetworks detected for T2C dataset included 200–250 genes. For T2D3 dataset, around two third of the subnetworks included 150–175 genes. In Figure S1, we have shown the distribution of the numbers of the genes included in the subnetworks, which are generated for different T2D datasets in our analysis. In this figure, we have observed that a similar distribution is observed between T2D1, T2D2 and T2D3 datasets. The distribution of the sizes of the subnetworks obtained from T2DC dataset is slightly different. The number of identified subnetworks including 151–200 genes is smaller for the T2DC dataset (197) compared to the number of identified subnetworks of this size in other datasets. The number of identified subnetworks including 201–250 genes is slightly higher for the T2DC dataset (562) compared to the number of identified subnetworks of this size in other datasets. In general, when the overall distribution of the subnetwork sizes are investigated, no big difference is observed. In the following steps for each identified subnetwork, functional enrichment analysis is carried out and hence, affected pathways are determined.

### 3.2. Dysregulated modules of T2D that are identified using network propagation (bottom-up approach)

Known T2D genes, collected in Ghiassian et al.’s (2015) study are used as seed genes to find dysregulated modules via expanding a module by adding other possible genes to the known disease gene clusters. This study indicated that seed proteins display unusual interaction patterns among each other. It enlightens the idea that the existence of disease specific modules is not by chance. Connectivity significance values are calculated for all neighbors of 73 known T2D disease associated seed genes. Afterwards, the node with the most significant interaction is added to the module and this iteration is repeated until 200 and 500 genes are included in a module. Then, functional enrichment procedure is performed on each of these two dysregulated modules (T2D_D200, T2D_D500).

### 3.3. Affected pathways of T2D

Based on the observation that genes almost always act cooperatively rather than independently, to facilitate the biological interpretation of high-throughput data, many different methods have been postulated to identify the biological pathways associated with a particular clinical condition under study. Here, to characterize this cooperative nature of genes and to elucidate the molecular mechanisms of T2D, we investigate the affected pathways of T2D and search for the potential failures in these wiring diagrams.

#### 3.3.1. Overrepresented pathways of T2D dysregulated modules (top-down approach)

**Figure 3:**
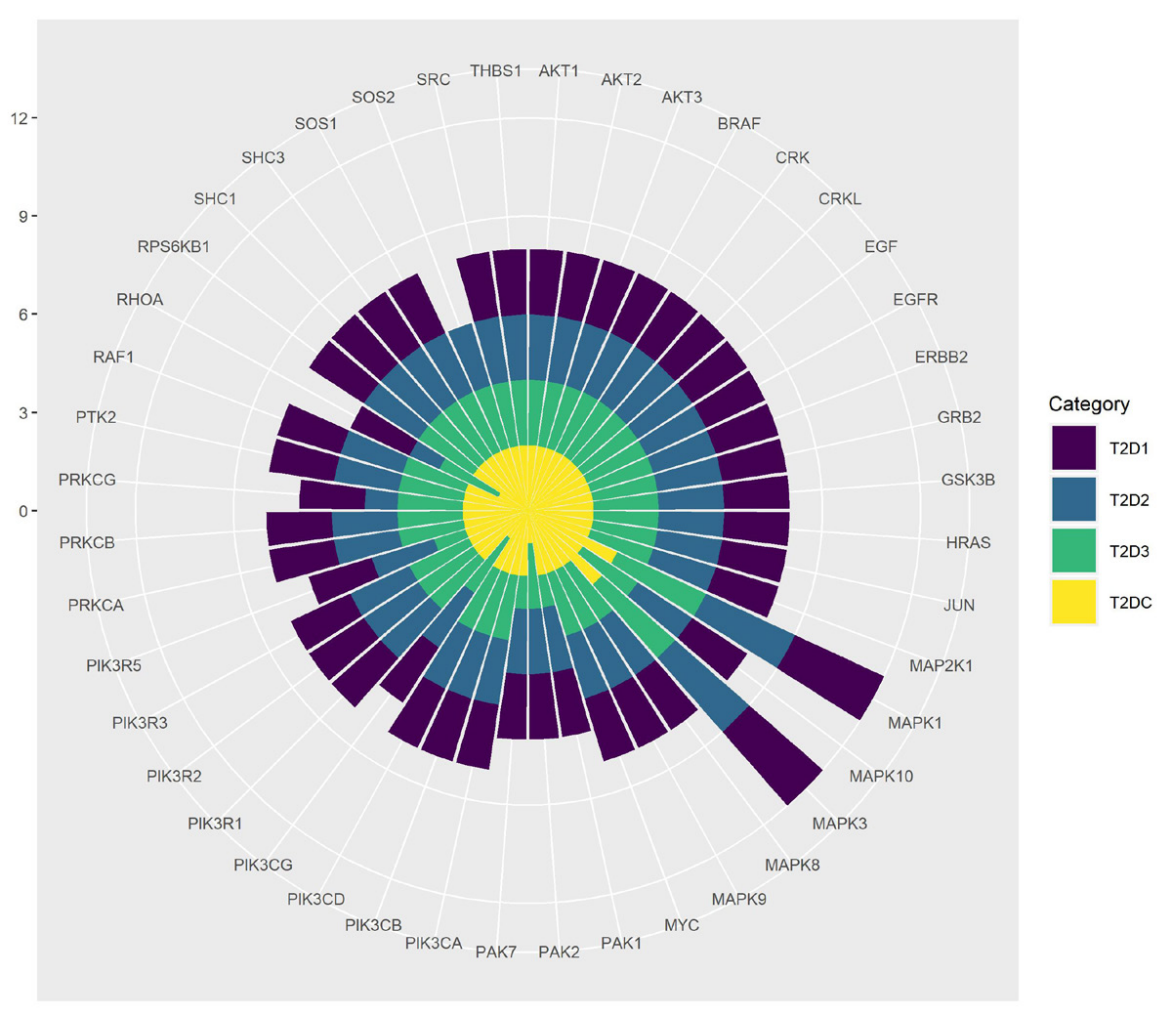
Highly targeted T2D genes that reside in five commonly identified pathways. Frequencies in different datasets are shown with different colors.

To detect possible pathogenic pathways related with T2D, the genes listed in each dysregulated module are compared with the genes included in KEGG pathways and the proportion of the module genes over all pathway-associated genes is calculated. Significantly affected KEGG pathways (pathways with corrected p-values < 0.05) for our defined dysregulated modules are appended to potentially significant pathway list of T2D disorder. Table 3 presents top 10 affected pathways that are found to be overrepresented in the dysregulated modules of T2DC dataset, and the rankings of these pathways in different datasets. The p-values of these identified pathways are listed in Table S1. Five of these pathways (shown in bold in Table 3) are also identified in all other T2D datasets. These shared pathways are spliceosome, focal adhesion, soluble N-ethylmaleimide-sensitive factor attachment protein receptor (SNARE) interactions in vesicular transport, transforming growth factor-β (TGF-β) signaling, and ErbB signaling pathways. Table 3 also displays the numbers of genes identified in different datasets for each pathway. Although these pathways are found to be affected in T2D in different datasets, for each dataset, different genes may be targeted. As shown in Table 3, for each affected pathway, the number of genes that are found in at least one dataset (union gene set for an identified pathway) can be up to 15% higher than the max number of targeted genes for an identified pathway. Hence, we report that although these 5 pathways are commonly affected in all metaanalysis GWAS datasets of T2D, different sets of genes may be targeted in each dataset. In our analysis, while all four metaanalysis GWAS data of T2D identifies the same 5 pathways, each dataset sheds light on slightly different sets of affected genes and it is worth to analyze these different sets of genes. We continued our analysis as following. Within these 5 commonly targeted pathways, the frequencies of the genes that are targeted in different datasets are further analyzed and shown in Figure 3. While AKT1, AKT2, AKT3, BCL2, BRAF, BTC, CCND2, CDKN2B, ERBB4, IGF1, LAMA1, PIK3CB, RAPGEF1, TGFB1, TNF, VEGFC, VTI1A genes are known to have a role in T2D development mechanism in DisGeNET (Piñero et al., 2019); the other genes that are highly represented in Figure 3 and in Table S2 can be potential T2D causing genes. The possible roles of these genes in T2D development are discussed in Section 4.

Additionally, the commonalities between the gold standard T2D pathways (Yoon et al., 2018) and the whole list of affected pathways that are enriched for the dysregulated modules of T2D1, T2D2, T2D3, T2DC datasets, are also studied. When the identified pathways are overlapped among all datasets and with the gold standard T2D pathway set (Yoon et al., 2018), 12 KEGG pathways are commonly observed. These pathways are valine, leucine and isoleucine degradation, cell cycle, glycolysis/gluconeogenesis, type II diabetes mellitus, fatty acid metabolism, JAK-STAT signaling, calcium signaling, insulin signaling, Wnt signaling, PPAR signaling, adipocytokine signaling, and Notch signaling pathways.

#### 3.3.2. Enriched pathways for the expanded modules of T2D seed genes (bottom-up approach)

Overrepresented pathways for expanded modules of T2D seed genes are identified with functional enrichment analysis. As shown in Table 4, the enrichment operation on T2D_D200 and T2D_D500 dysregulated modules (including 200 and 500 genes) resulted in 41 and 84 significant pathways, respectively.

#### 3.3.3. The pathways that are identified using Pascal algorithm on T2D GWAS metadata

The Pascal algorithm, as explained in subsection 2.2.9, is used to find potentially affected pathways for T2D1, T2D2, and T2D3 data sets. Firstly, gene and pathway scores from SNP-phenotype association summary statistics are computed for each dataset. Secondly, the calculated scores of affected pathways for each dataset are combined with Fisher’s method, and consequently, 38 KEGG and 46 Reactome pathways are detected.

**Figure 4:**
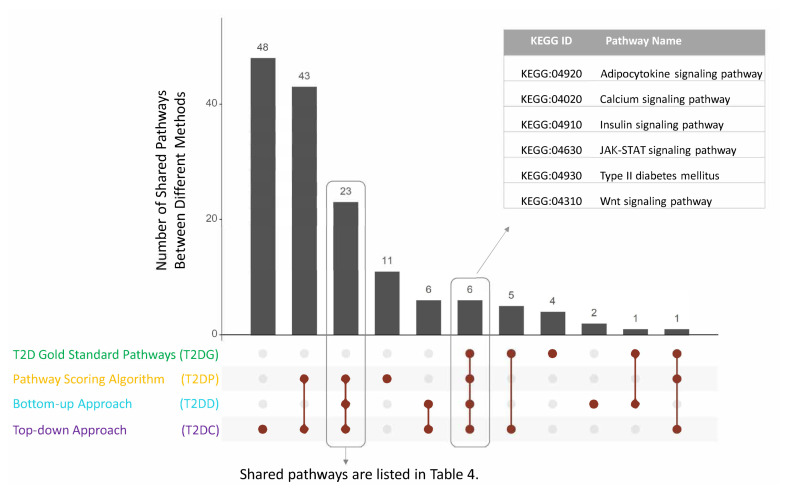
Comparison of the affected pathways that are identified using top-down approach, bottom-up approach and Pascal, and the gold-standard pathways of T2D.

The affected pathways of T2D using top-down approach, bottom-up approach and Pascal algorithm are described in subsections 3.3.1, 3.3.2, 3.3.3, respectively. Once we detect the affected pathways of T2D using three different approaches, we also analyzed the commonalities among the results of these approaches. The commonly identified KEGG pathways of T2D are listed in Table 4 with their rankings and p-values in different approaches; and visualized in Figure 4. Six of these affected pathways, which are highlighted in bold in Table 4 and shown in Figure 4 refers to the gold standard KEGG pathways of T2D reported in Yoon et al.’s (2018) study. These six commonly identified KEGG pathways are type II diabetes mellitus, Insulin signaling, JAK-STAT signaling, Calcium signaling, Adipocytokine signaling, and Wnt signaling pathways. Additionally, we have commonly identified GnRH signaling pathway, pancreatic cancer, adherens junction, ErbB signaling pathway, Phosphatidylinositol signaling system, neurotrophin signaling pathways in all three methods. Although these pathways (highlighted in italic in Table 4) are not included in the gold standard pathways of T2D, they could have potential role in T2D development mechanisms, as discussed in detail in Section 4.

#### 3.3.4. Affected pathway subnetworks and pathway clusters of T2D

We hypothesized that similar to the dysregulated modules of proteins, dysregulated modules of pathways have a role in disease development mechanisms. In order to identify affected pathway subnetworks of a disease; we proposed a methodology, as shown in Figure 2. Instead of a PPI network, this method requires a pathway network as the baseline. Here, we utilized the 288 human KEGG pathways as a reference, for the generation of this biological network. To establish a pathway network, firstly, we examined the relationships between the genes and the biological pathways, as explained in subsection 2.2.7. In this study, we stored these relationships in a gene-term matrix, which is a binary matrix with dimensions 6881 × 288, representing the number of individual genes in all pathways, and the number of pathways, respectively. Secondly, the relationships between the pathways are analyzed, as explained in subsection 2.2.7. For this purpose, kappa statistics was used to determine the relationships between pathways, and a term-term matrix (of size 288 × 288), was formed by using the previously obtained gene-term matrix. Thirdly, to identify interrelated pathways, we experimented with different cutoff values of kappa scores. The sizes of the networks that are created with different threshold values are presented in Table S3. Since the node to edge ratio in the human PPI network is approximately 1 to 10, the kappa score threshold value is selected as 0.15 in this study and finally, a human pathway network including 288 pathways (nodes) and 2976 interrelations (edges) is created.

Active subnetwork identification algorithms require a biological network and the significance values of the nodes, e.g., the p-values of the genes obtained from microarray studies, indicating the significance of a gene, in terms of the expression levels differing between two experimental conditions. Here, while our biological network is selected as our generated pathway network, significance values of the nodes are selected as the corrected hypergeometric test p-values, indicating the importance of the pathway for T2D. Following the methodology proposed in Figure 2, for all T2D datasets, only one affected pathway subnetwork exceeded the predefined subnetwork score, as summarized in Table S3. As the node and edge numbers of these identified pathway subnetworks could be inspected from Table S3, it could be observed that the nodes are severely connected to each other in the identified pathway subnetworks. Therefore, these four identified pathway subnetworks (for four different datasets) were further grouped into subcategories as explained in subsection 2.2.8, and the affected pathway clusters of T2D are obtained for each dataset. As shown in Table S4, for T2D1, T2D2, T2D3, T2DC datasets, 7, 9, 7, and 8 affected pathway clusters are identified, respectively. Numbers of nodes (pathways) included in each cluster and the scores of each pathway cluster can be found in Table S4. When the obtained results are analyzed, it is seen that the initial pathway subnetwork, which is severely connected with each other and has more than 50 nodes is successfully divided into smaller disease related subnetworks. This can be considered as a proof of the effectiveness of the developed method. The highest scoring pathway cluster of T2D1, T2D2, T2D3, T2DC datasets included 38, 34, 35 and 35 pathways, respectively. For each dataset, the representative networks of the identified pathway clusters are shown in Figure S2. In this figure, while the node IDs indicate the corresponding KEGG pathway IDs, the edges indicate that the number of common genes between two pathways is more than a predefined threshold. When we analyze the commonalities among these pathways, we observed that 27 of these pathways were commonly identified in T2D1, T2D2, T2D3, T2DC datasets. The details of these commonly identified pathways within pathway clusters of different datasets are given in Table 5.

**Figure 5:**
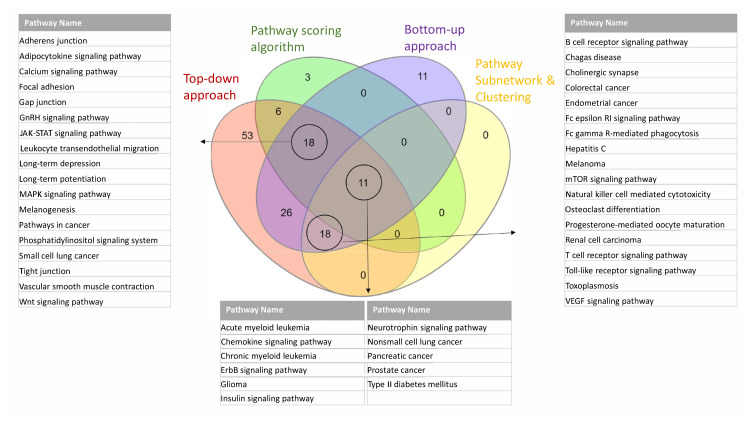
Commonalities between the affected pathways that are identified using four different approaches, namely, i) top-down approach, ii) bottom-up approach, iii) Pascal, iv) pathway subnetworks and pathway clusterings.

Via analyzing multiple association studies of T2D with four different approaches, namely, i) top-down approach, ii) bottom-up approach, iii) Pascal algorithm, iv) pathway subnetworks and pathway clusterings; we presented our findings in subsections 3.3.1, 3.3.2, 3.3.3 and 3.3.4, respectively. Among these different approaches, we summarized the commonalities between the affected pathways in Figure 5. In addition to the well-known T2D pathways (e.g., insulin signaling pathway, type II diabetes mellitus pathway), additional pathways are commonly identified by at least three of the four approaches. These pathways are listed in Figure 5.

In Table 6, we provide a consensus list of T2D pathways to follow up on. Among these pathways while 11 pathways are identified by all four approaches (as shown in Figure 5), 12 pathways are identified by our proposed top-down approach in all three metaanalysis GWAS data of T2D (as presented in subsection 3.3.1) and also included in the gold standard pathways of T2D (Yoon et al., 2018); and 5 pathways are commonly identified in the top 10 lists of top-down approach on all three metaanalysis GWAS data of T2D (as presented in Table 3). The 11 pathways that are commonly detected in all four approaches are acute myeloid leukemia, chemokine signaling pathway, chronic myeloid leukemia, ErbB signaling pathway, glioma, insulin signaling pathway, neurotrophin signaling pathway, nonsmall cell lung cancer, pancreatic cancer, prostate cancer, type II diabetes mellitus. In order to reduce the potential redundancy within the consensus T2D pathways, we calculated the Kappa scores between each pair of consensus T2D pathways. Kappa score statistics quantitatively measures the degree of the agreement between the pathways, via comparing the amount of similar genes. In Table S5, we presented these scores along with the numbers of common genes between consensus T2D pathway pairs. We observed in Table S5 that 7 pathways (Chronic myeloid leukemia, Nonsmall cell lung cancer, acute myeloid leukemia, ErbB signaling pathway, pancreatic cancer, prostate cancer, glioma) among 25 consensus T2D pathways share similar genes (their pairwise Kappa scores are higher than 0.5) and all these 7 pathways are cancer related pathways. Additionally, we have visualized the commonalities among our consensus T2D pathways (the Kappa scores) in a heatmap in Figure S3. In this figure we observed that the pairwise combinations of the abovementioned 7 pathways have red, purple, blue colors (Kappa scores higher than 0.5) in the heatmap. Hence, we simplified Table 6 via merging these pathways into a single cluster and we kept other consensus T2D pathways that are driven by unique gene signatures.

**Figure 6:**
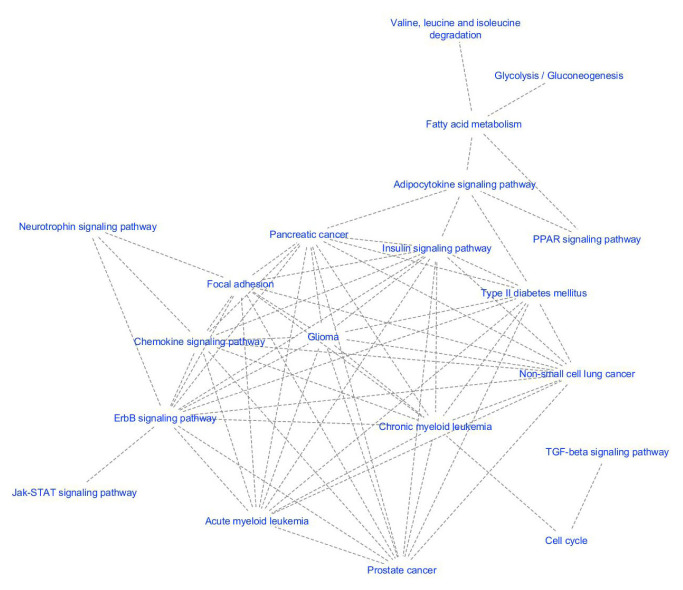
The relationship between the consensus KEGG pathways of T2D (pathway list in Table 6). The edges between the pathways are defined by their shared genes (calculated using the Kappa score as presented in Supplementary Figure 3 and Supplementary Table 5).

In order to get a better idea about the relationships between the T2D risk pathways in our consensus list, we generated pathway relationship network in Figure 6. In Figure 6, the edges between the pathways are defined by their shared genes (calculated using the Kappa score as presented in Figure S3 and Table S5). As shown in Figure 6 and Table S5, there are almost no common genes between Wnt signaling, calcium signaling, Notch signaling, spliceosome, and SNARE interactions in vesicular transport pathways (the pair-wise Kappa scores between these pathways are less than the predefined threshold). Hence, they are kept as individual nodes in the network of consensus T2D pathways (Figure 6). In Figure 6, we also color-coded cancer-related pathways as grey, signaling pathways as orange and other pathways as green.

### 3.4. Shared T2D subnetworks and pathways among different GWAS metadata

#### 3.4.1. Comparative evaluation of identified T2D subnetworks for each dataset

The identified T2D1, T2D2, T2D3 and T2DC subnetworks (as explained in subsection 3.1, and summarized in Figure S1) are compared in a pairwise manner to assess the shared information among them. Firstly, for each x, y pairs of T2D1, T2D2, T2D3 and T2DC datasets, each identified subnetwork of T2D_x_ dataset and T2D_y_ dataset are compared in gene level and a contingency table of T2D_x_/T2D_y_, as shown in Table 1, is created. In this contingency table, each value of n_ij_ represents the shared gene counts between the ith subnetwork of T2D_x_ dataset and the jth subnetwork of T2D_y_ dataset. Secondly, based on this table, the entropy values H(T2D_x_), H(T2D_y_) and the mutual information values I(T2D_x_, T2D_y_) are computed for each x, y dataset pair. Thirdly, normalized mutual information (NMI) is calculated as explained in subsection 2.2.10. This procedure is repeated for all pairwise combinations of the T2D datasets. Hence, similarity scores (NMI_SUM_) are calculated between all pairs of datasets. The presented heatmap in Figure S4 illustrate the similarities of datasets according to the strength of the NMI_SUM_ score. As illustrated in Figure S4A, T2D1, T2D2, T2D3 and T2DC subnetwork similarities are resulted in range [0, 0.01]. While the highest similarity score of 0.0073 is obtained for T2D2-T2D3 dataset pair, the lowest score of 0.0060 is obtained for T2D1-T2DC dataset pair. Accordingly, while the darker colors indicate higher correlation, lighter colors indicate smaller correlation in the heatmap of Figure S4A. NMI_SUM_ scores in the diagonals of the heatmap are “whitened” for clearer visibility of the other NMI_SUM_ values.

#### 3.4.2. Comparative evaluation of identified T2D pathways for each dataset

Shared information among different methodologies (subnetwork identification, as presented in subsection 2.2.5.1 and bottom-up approach, as presented in subsection 2.2.5.2) and different T2D metadatasets, are also evaluated in terms of the identified T2D pathways. The same functional enrichment analysis is applied on the subnetworks and dysregulated modules, as explained in subsection 2.2.6. In addition to the identified pathways of T2D1, T2D2, T2D3 and T2DC datasets, the pathways identified from T2D_D200 and T2D_D500 gene sets are also evaluated here. Firstly, for each x, y pairs of T2D1, T2D2, T2D3, T2DC, T2D_D200 and T2D_D500, each identified pathway of T2D_x_ dataset and T2D_y_ dataset are compared in terms of their common genes and a contingency table of T2D_x_/T2D_y_ is created, as shown in Table 1. In this contingency table, each value of n_ij_ represents the shared gene counts between the ith identified pathway of T2D_x_ dataset and the jth identified pathway of T2D_y_ dataset. Secondly, based on this table, the entropy values H(T2D_x_), H(T2D_y_) and mutual information values I(T2D_x_, T2D_y_) are computed for each x, y dataset pair. Thirdly, normalized MI is calculated as explained in subsection 2.2.10. This procedure is repeated for all pairwise combinations of the T2D datasets. Hence, similarity scores (NMI_SUM_) are calculated between all pairs of datasets, in terms of overrepresented pathways. In terms of the identified pathways, Figure S4B illustrates the similarity levels of the T2D1, T2D2, T2D3, T2DC, T2D_D200 and T2D_D500, in the range of [0–0.1]. While a maximum NMI_SUM_ score of 0.0658 is achieved for T2D1-T2D3 pair, a minimum NMI_SUM_ score of 0.016 is obtained for T2DC-T2D_D200 pair. Accordingly, while the darker colors indicate higher correlation, lighter colors indicate smaller correlation in the heatmap of Figure S4B. NMI_SUM_ scores in the diagonals of the heatmap are “whitened” for clearer visibility of the other NMI_SUM_ values.

## 4. Discussion

GWASs of T2D have significantly accelerated the discovery of T2D–associated loci (Bonnefond and Froguel, 2015; Adeyemo et al., 2015; Scott et al., 2017; Meyre, 2017; Liu et al., 2017). Although the identified T2D-risk variants including 243 loci and 403 distinct association signals exhibit a potential for clinical translation, the genome-wide chip heritability explains only 18% of T2D risk (Bonàs-Guarch et al., 2018; Mahajan et al., 2018a; Xue et al., 2018). Traditional GWASs focus on top-ranked SNPs and discard all others except ‘the tip of the iceberg’ SNPs. Such GWAS approaches are only capable of revealing a small number of associated functions. In this regard, even though GWASs are a compelling method to detect disease-associated variants, it does not directly address the biological mechanisms underlying genetic association signals, and hence, the development of novel post-GWAS analysis methodologies is needed (Lin et al., 2017; Gallagher and Chen-Plotkin, 2018; Erdmann and Zeller, 2019). In this respect, to enlighten the molecular mechanisms of T2D development, here we proposed a method that perform protein subnetwork, pathway subnetwork and pathway cluster level analyses of the SNPs that are found to be mildly associated with T2D in multiple association studies. In other words, to achieve a coherent comprehension of T2D molecular mechanisms, the proposed network and pathway-based solution conjointly analyzes three metaanalyses of GWAS, which are conducted on T2D.

The baseline of our study is built on the interactions of T2D related proteins since the proteins act as the functional base units of the cells and construct the frameworks of cellular mechanisms. Protein network structure helps us to gain a collective insight about the biological systems. At the mesoscopic level of these protein networks, active modules are the potential intermediate building blocks between individual proteins and the global interaction network. Dysregulation of these modules are considered to have a role in disease development mechanisms. Hence, the identification of dysregulated modules of T2D helps us to understand the fundamental molecular characteristics of T2D and to discover new candidate disease genes having a role in the regulation of T2D related pathways. In this context, for each analyzed T2D GWAS metaanalysis dataset (where the characteristics of each dataset is summarized in Table 2), 800 to 1000 dysregulated modules, including 150 to 250 genes are detected using a top-down approach, as explained in subsection 2.2.5.1. As outlined in Figure 1, these modules are functionally enriched and the pathways that have a potential effect on T2D development are identified. As presented in Table 3, among the top 10 affected T2D pathways of T2DC datasets, 5 pathways are commonly overrepresented for the dysregulated modules of T2D1, T2D2, T2D3, T2DC datasets. These five shared pathways are spliceosome, focal adhesion, SNARE interactions in vesicular transport, TGF-β signaling, and ErbB signaling pathways. Spliceosome pathway has a role in the regulation of alternative splicing in insulin resistance cases by aberrantly spliced genes like *ANO1, GCK, SUR1, VEGF* (Costantini et al., 2011; Schmid et al., 2012; Dlamini et al., 2017). Focal adhesion pathway is complementary in regulation of insulin signaling pathway. Via controlling adipocyte survival, focal adhesion kinases (FAK) regulate insulin sensitivity (Luk et al., 2017). SNARE protein contributes to fusion mechanism of insulin secretory vesicles (Xiong et al., 2017). The study conducted by Boström et al. demonstrated that total skeletal muscle SNARE protein SNAP23 and SNARE related Munc18C protein levels are higher in patients with T2D, which are also correlated with markers of insulin resistance (Boström et al., 2010). TGF-β signaling pathway has role in inflammation by cytokines such as interleukins, tumor necrosis factors, chemokins interferons, transforming growth factors (TGF). Insulin enhances TGF-β receptors in fibroblasts and epithelial cells. Herder et al. documented that high levels of antiinflammatory immune mediator TGF-β1 are correlated with T2D (Herder et al., 2009). TGF-β signaling pathway is also shown to have a crucial role in extracellular matrix accumulation in diabetic nephropathy (Kajdaniuk et al., 2013). Akhtar et al. showed that the dysregulation of epidermal growth factor receptor family (ErbB) triggers vascular dysfunction stimulated by hyperglycemia in T2D (Akhtar et al., 2015). Other dual role of ErbB protein family included diabetes triggered cardiac dysfunction (Akhtar and Benter, 2013). Within these five pathways, we identified additional genes that are highly represented in the generated subnetworks of all three datasets (as shown in Figure 3 and in Table S2). Among these genes, *CRK, CRKL, EGF, EGFR, ERBB2, GRB2, GSK3B, HRAS, JUN, MAP2K1, MAPK1, MAPK10, MAPK3, MAPK8, MAPK9, MYC, PAK1, PAK2, PAK7, PIK3CA, PIK3CD, PIK3CG, PIK3R1, PIK3R2, PIK3R3, PIK3R5, PRKCA, PRKCB, PRKCG, PTK2, RAF1, RHOA, RPS6KB1, SHC1, SHC3, SOS1, SOS2, SRC, THBS1* genes can be potential T2D causing genes. In insulin uptake mechanism, insulin binds to its insulin receptor and intracellular signaling molecules are induced. Phosphatidylinositol 3-kinase (*PI3K*) is one of the highly represented genes in our study and it stimulates glucose uptake into muscle cells. PI3Ks consist of regulatory and catalytic subunits. Among the overrepresented genes in our study, *PIK3R1, PIK3R2, PIK3R3* and *PIK3CA, PIK3CD* encode these subunits respectively (Maffei et al., 2018). Excessive free fatty acid accumulation in skeletal muscle cells impairs PI3K/AKT signaling, causes insulin resistance, and eventually leads to obesity and T2D (Huang et al., 2018). Another overrepresented gene *GSK3ß* is a key kinase and plays a causative role in impairment of insulin signaling by degradation of insulin receptor substrate (IRS1) (Leng et al., 2010). Leng et al. also stated that p-21 activated kinase (PAK) signaling have role in glucose homeostasis and cancer. PAK2 and PAK7 proteins from PAK family are highlighted in our study and they are potential molecular targets in T2D. In the same study (Leng et al., 2010), PAK interacting partners are reported as *SOS1, SHC1, EGFR, GRB2, GSK3ß* and *PIK3R1*, and these genes are identified in our highly represented genes list.

While identifying active subnetworks of T2D, in addition to the top-down approach (as discussed above), we also applied bottom-up approach as explained in subsection 2.2.5.2. Overrated pathways of i) top-down approach (T2DC), ii) bottom-up approach (T2D_D200, T2D_D500), and iii) Pascal (T2D_P) are comparatively evaluated. Among these pathways, type II diabetes mellitus, calcium, insulin, Wnt, adipocytokine, JAK-STAT signaling pathways (shown in bold in Table 4) overlap with gold standard pathways of T2D (Yoon et al., 2018). Additionally, the pathways that are shown in italic in Table 4 have support from the literature as following. The study conducted by (Berntorp et al., 2013) reported that T2D patients express antibodies against gonadotropin-releasing hormone GnRH in serum. (De Souza et al., 2016) stated T2D as prognostic and risk factor for pancreatic cancer. Houtz et al. (2016) reported that paracrine neurotrophin signaling have a role in insulin secretion between pancreatic vascular system and beta cells, which is triggered by glucose. Ono et al. (2001) stated that phosphatidylinositol signaling system including PTEN (phosphatase and tensin homologue deleted on chromosome 10) and PI3K (phosphoinositide3-kinase) proteins regulate glucose homeostasis and insulin metabolism. In a study performed by (Dissanayake et al., 2018), cadherin mediated adherens junction proteins are shown to have a potential regulation role in insulin secretion mechanism by controlling vesicle traffic in cell. Via studying different GWAS metaanalyses, Schierding et al. indicated the spatial connection of *CELSR2–PSRC1* locus with *BCAR3*, which is part of the insulin signaling pathway (Schierding and O’Sullivan, 2015). The post-GWAS study conducted by Liu et al. (2017) identified T2D risk pathways. Among these pathways, type II diabetes mellitus, calcium signaling pathway, cell cycle, pancreatic cancer, MAPK signaling pathway, chemokine signaling pathway, Tight junction pathways were also identified in our study (p < 0.05). Another study performed by Perry et al. (2009) analyzed T2D GWAS data and reported that Wnt signaling pathway, olfactory transduction, galactose metabolism, pyruvate metabolism, type II diabetes, TGF-signaling pathways are associated with T2D. Wnt signaling and type II diabetes pathways are overlapped with our findings, as shown in Table 4. The analysis of T2D WTCCC GWAS dataset by (Zhong et al., 2010) indicated 22 affected pathways in T2D. Among these pathways, tight junction, phosphatidylinositol signaling system, pancreatic cancer, adherens junction, calcium signaling pathway are replicated in our study, as shown in Table 4.

### 3.1. Systematic assessment of the proposed pathway identification methods: ability to identify the gold standard pathways of T2D

Nguyen et al. proposed that the validation of a pathway analysis method is realized via evaluating its ability to identify the target pathway describing the related mechanism of the condition studied (Nguyen et al., 2019). For this purpose, they collected data sets related to conditions that already have an associated KEGG pathway (i.e. target pathway). They assumed that a perfect method should be able to identify the target pathway as significantly impacted and rank it on top. They applied different pathway analysis methods on each of those data sets and reported the ranks and the p-values of target pathways. Inspired by their approach, here we compared the performances of three different pathway identification methods on four different T2D GWAS metaanalysis datasets (T2D1, T2D2, T2D3, T2DC).

**Figure 7:**
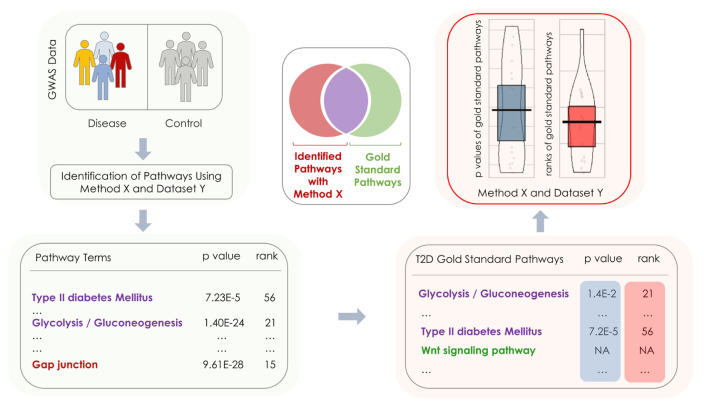
A workflow to evaluate a pathway analysis method’s performance, based on its ability to identify gold standard KEGG pathways of T2D.

**Figure 8:**
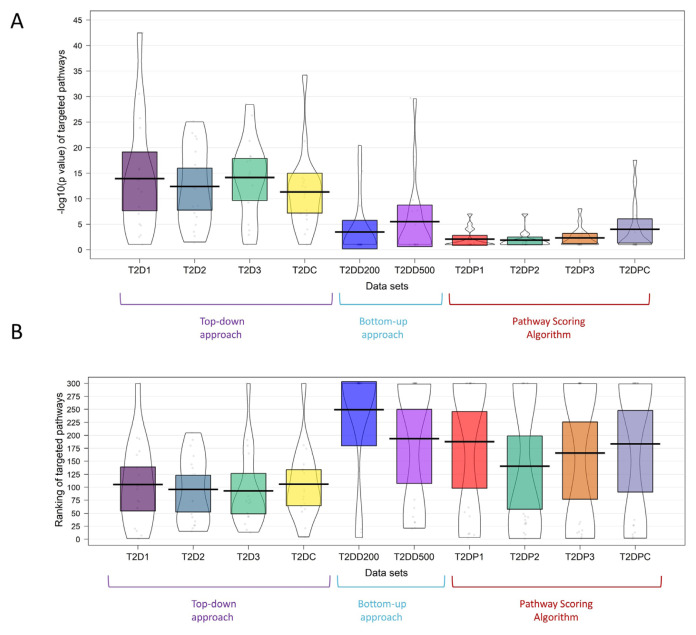
(A) p-values and (B) the ranks of gold standard KEGG pathways of T2D, derived by top-down approach on three different datasets; bottom-up approach; Pascal.

In Figure 7, we summarized our workflow to compare the pathway identification methods. As illustrated in Figure 7, different methods and datasets are evaluated based on their ability to rank the target pathways of T2D. Each method and dataset produces lists of ranks and p-values for the target pathways of T2D, which are then used to assess the method’s performance. In Figure 8, the resulting ranks and p-values of the target pathways are plotted in violin plots. While the horizontal axis shows the method and the dataset, the vertical axis in Figure 8A represents the ranks, and the vertical axis in 8B corresponds to the −log10(p-values) of the target pathways. As target pathways, we used the gold standard pathways of T2D (Yoon et al., 2018).

We perform a comparison between the ranks and the p-values of the gold standard T2D pathways obtained by top-down approach, bottom-up approach and Pascal algorithm on three datasets. As shown in Figure 8A, for the 17 gold standard pathways of T2D, our post-GWAS analysis methodology (top-down approach) yielded in higher –log10 (p-values) on all four metaanalysis GWAS datasets of T2D than bottom-up approach (DIAMOnD algorithm based on network propagation (Ghiassian et al, 2015), shown as T2D200 and T2D500 in figure) and Pascal algorithm (Lamparter et al, 2016), shown as T2DP1, T2DP2, T2DP3, T2DPC in figure). As shown in Figure 8B, our post-GWAS analysis methodology (top-down approach) identified the 17 gold standard pathways of T2D in lower rankings on all four metaanalysis GWAS datasets of T2D than bottom-up approach (DIAMOnD algorithm based on network propagation (Ghiassian et al, 2015), shown as T2D200 and T2D500 in figure) and Pascal algorithm (Lamparter et al, 2016), shown as T2DP1, T2DP2, T2DP3, T2DPC in figure).

**Figure 9:**
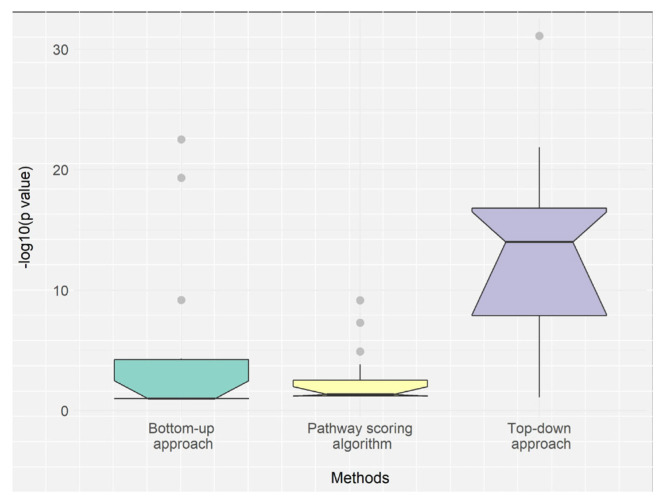
The performances of top-down approach, bottom-up approach, Pascal in term of –log10 (p-values) of gold standard KEGG pathways of T2D. We collect all the p-values that are obtained for gold standard pathways of T2D using different approaches and different datasets in Figure 8 and categorize them accordingly into three groups. The higher –log10 (p-values) indicate better performance.

Additionally, we conduct a higher level comparison between three different approaches. The median p-values obtained by using top-down approaches are also significantly lower (Wilcoxon p-value = 6.289 E–4) than those of the bottom-up approaches; and significantly lower (Wilcoxon p-value = 6.948 E–5) than those of the pathway scoring algorithm (Figure 9). These results suggest that top-down approaches perform superior to the bottom-up approach and Pascal algorithm.

Within the gold standard KEGG pathways of T2D (Yoon et al., 2018), the frequencies of the genes that are targeted in different datasets are also analyzed. In Figure S5, we present the frequencies of the highly targeted T2D genes that reside in gold standard KEGG pathways of T2D. The different colors in Figure S5 refer to the frequencies in different datasets. While some of the genes that are listed in Table S6 are known to have a role in T2D development mechanism in DisGeNET (Piñero et al., 2019); the other genes that are highly represented in Figure S5 and in Table S7 can be potential T2D causing genes. *ALDH1B1* as one of these highly represented genes belongs to aldehyde dehydrogenase gene family. This gene encodes mitochondrial ALDH1B1 protein which regulates progenitor cells in mouse pancreas development. Studies showed that loss of function of this enzyme induces deficiency in mouse ß-cells and upregulation of ALDH1B1 enzyme was identified in human pancreatic cancer (Mameishvili et al., 2019). Other overrepresented genes encoding EP300 and CREBBP transcriptional coactivators contribute to development and maintain proper functioning of ß-cells. Functional inactivation of either p300 or CBP in mice lead to glucose intolerance and reduction ß-cells mass (Wong et al., 2018).

Using the mutual information based on the shared genes, the identified protein subnetworks and the affected pathways of each dataset were compared. While the NMISUM subnetwork scores range from 0 to 0.01, NMISUM pathway scores range from 0 to 0.1 (as shown in Figure S4). Hence, we show that while the subnetwork level analyzes increase the degree of irregularity, pathway level evaluation of different T2D GWAS metadata and different methodologies (top-down vs. bottom-up approach) resulted in higher levels of conservation and yielded in more interpretable outcome.

While the type II diabetes mellitus pathway is identified in the later rankings for T2D1, T2D2, T2D3, and T2DC GWAS datasets (as shown in Table 5), the incorporation of the generated pathway network information helped us to prioritize this pathway. This pathway is found in the highest scoring pathway cluster of each dataset. Since the pathways are strongly interrelated, our proposed approach created a pathway network, and identified affected pathway subnetworks and pathway clusters using multiple association studies, which are conducted on T2D. Our approach is based on both significance level of an affected pathway and its topological relationship with its neighbor pathways.

## 4. Conclusion

In conclusion, the availability of T2D GWAS metadata and new analytical methods has provided opportunities to bridge the knowledge gap from sequence to consequence. In this study, the collective effects of T2D–associated variants are inspected using network and pathway-based approaches, and the prominent genetic association signals related with T2D biological mechanisms are revealed. We presented a comprehensive analysis of three different T2D GWAS metadata at protein subnetwork, pathway, and pathway subnetwork levels. To explore whether our results recapitulate the pathophysiology of T2D, we performed functional enrichment analysis on the dysregulated modules of T2D. In addition to our analysis of the shared information among different datasets in terms of subnetworks, we also analyzed the shared information in terms of the identified T2D pathways. The identified pathway subnetworks, pathway clusters and affected genes within these pathways helped us to illuminate T2D development mechanisms. We hope the affected genes and variants within these identified pathway clusters help geneticists to generate mechanistic hypotheses, which can be targeted for large-scale empirical validation through massively parallel reporter assays at the variant level; and through CRISPR screens in appropriate cellular models, and through manipulation in in vivo models, at the gene level.

Supplemental Figure 1 Numbers of genes included in the identified (A) 983 subnetworks for T2D1, (B) 903 subnetworks for T2D2, (C) 940 subnetworks for T2D3, and (D) 813 subnetworks for T2DC datasets.

Supplemental Figure 2 The representative networks of the highest scoring pathway clusters of (A) T2D1, (B) T2D2, (C) T2D3, (D) T2DC datasets, including 38, 34, 35 and 35 pathways, respectively.

Supplemental Figure 3 The commonalities among our consensus T2D pathways (based on the Kappa scores). While red, purple and blue colors represent higher commonalities between the genes of a pathway pair and also higher Kappa scores; the green color represents less commonality between the genes of a pathway pair and also lower Kappa score for a pathway pair in the heatmap. The white color represents that none of the genes or very small numbers of genes are common between the genes of a pathway pair.

Supplemental Figure 4 Shared information comparison among different datasets in terms of (A) identified T2D subnetworks, and (B) identified pathways via normalized mutual information (NMISUM). While the darker colors indicate higher correlation, lighter colors indicate smaller correlation. NMISUM scores in the diagonals of the heatmap are “whitened” for clearer visibility of the other NMISUM values.

Supplemental Figure 5 Highly targeted T2D genes that reside in gold standard KEGG pathways of T2D. Frequencies in different datasets are shown with different colors.

**Supplementary Table 1:** The p-values and the rankings of the top 10 affected pathways of four datasets using top-down approach. Among these pathways, 5 pathways (shown in bold) are commonly overrepresented for the dysregulated modules of T2D1, T2D2, T2D3, T2DC datasets.

	p-values				Rank				# of genes identified in different datasets (DD)				# of genes found in at least one dataset (Union)	# of genes in pathway (GiP)	Percent of identified genes in pathways	
															max (DD) / GiP	Union / GiP
KEGG term	T2DC	T2D1	T2D2	T2D3	T2DC	T2D1	T2D2	T2D3	T2DC	T2D1	T2D2	T2D3				
Spliceosome	8.55E-39	3.26E-27	6.95E-30	3.10E-41	1	15	8	5	65	62	75	**85**	104	127	0.66	0.81
Focal adhesion	7.032E-38	1.80E-30	3.82E-42	1.97E-54	2	10	1	1	**150**	135	146	146	172	200	0.75	0.86
SNARE interactions in vesicular transport	1.98E-35	1.37E-37	8.16E-33	5.41E-44	3	3	5	4	31	30	29	**32**	34	36	0.88	0.94
Valine leucine and isoleucine degradation	5.97E-35	3.26E-43	6.39E-20	3.34E-29	4	1	34	13	36	36	35	**37**	41	44	0.84	0.93
Purine metabolism	7.60E-34	5.35E-43	4.92E-12	1.29E-45	5	2	83	3	54	57	34	**92**	99	166	0.55	0.59
Dopaminergic synapse	3.26E-33	1.04E-20	9.48E-32	6.80E-34	6	37	7	9	107	107	**110**	103	119	130	0.84	0.91
TGF-beta signaling pathway	5.03E-29	8.70E-32	5.61E-34	3.23E-28	7	6	3	15	62	**64**	**64**	58	75	84	0.76	0.89
ErbB signaling pathway	1.59E-28	4.64E-31	1.00E-29	1.46E-37	8	8	9	7	**84**	80	82	81	85	87	0.96	0.97
Chemokine signaling pathway	5.23E-28	1.47E-21	1.01E-23	2.97E-19	9	33	20	39	107	**139**	111	129	163	189	0.73	0.86
Glutamatergic synapse	3.47E-27	1.97E-20	1.94E-29	3.03E-28	10	38	10	14	81	86	**88**	87	101	126	0.69	0.80

**Supplementary Table 2:** Potential T2D causing genes that are highly represented in five shared pathways among the top 10 affected pathways of four datasets using top-down approach.

						Spliceosome	ErbB signaling pathway	SNARE interactions in vesicular transport	TGF-beta signaling pathway	Focal adhesion
Gene	Total Frequency	T2D1	T2D2	T2D3	T2DC	hsa03040	hsa04012	hsa04130	hsa04350	hsa04510
AKT1	8	2	2	2	2		✓			✓
AKT2	8	2	2	2	2		✓			✓
AKT3	8	2	2	2	2		✓			✓
BRAF	8	2	2	2	2		✓			✓
CRK	8	2	2	2	2		✓			✓
CRKL	8	2	2	2	2		✓			✓
EGF	8	2	2	2	2		✓			✓
EGFR	8	2	2	2	2		✓			✓
ERBB2	8	2	2	2	2		✓			✓
GRB2	8	2	2	2	2		✓			✓
GSK3B	8	2	2	2	2		✓			✓
HRAS	8	2	2	2	2		✓			✓
JUN	8	2	2	2	2		✓			✓
MAP2K1	8	2	2	2	2		✓			✓
MAPK1	12	3	3	3	3		✓		✓	✓
MAPK10	8	2	2	2	2		✓			✓
MAPK3	12	3	3	3	3		✓		✓	✓
MAPK8	8	2	2	2	2		✓			✓
MAPK9	8	2	2	2	2		✓			✓
MYC	8	2	2	2	2		✓		✓	
PAK1	7	2	2	1	2		✓			✓
PAK2	7	2	2	2	1		✓			✓
PAK7	7	2	2	1	2		✓			✓
PIK3CA	8	2	2	2	2		✓			✓
PIK3CB	8	2	2	2	2		✓			✓
PIK3CD	8	2	2	2	2		✓			✓
PIK3CG	7	2	2	2	1		✓			✓
PIK3R1	8	2	2	2	2		✓			✓
PIK3R2	8	2	2	2	2		✓			✓
PIK3R3	8	2	2	2	2		✓			✓
PIK3R5	7	2	2	1	2		✓			✓
PRKCA	8	2	2	2	2		✓			✓
PRKCB	8	2	2	2	2		✓			✓
PRKCG	7	2	1	2	2		✓			✓
PTK2	8	2	2	2	2		✓			✓
RAF1	8	2	2	2	2		✓			✓
RHOA	6	2	1	2	1		✓		✓	✓
RPS6KB1	8	2	2	2	2		✓		✓	
SHC1	8	2	2	2	2		✓			✓
SHC3	8	2	2	2	2		✓			✓
SOS1	8	2	2	2	2		✓			✓
SOS2	6	0	2	2	2		✓			✓
SRC	8	2	2	2	2		✓			✓
THBS1	8	2	2	2	2				✓	✓

**Supplementary Table 3:** Node – edge relationships in the generated pathway networks and affected pathway subnetworks.

Sizes of the generated pathway networks for different threshold values		
Threshold values ( ≥ )	# of nodes	# of edges
0	288	82944
1.21E-5	288	10904
0.05	288	6806
0.1	288	4617
0.15	288	2976
0.2	288	1866
0.25	288	1321
Sizes of the generated highest scoring pathway subnetworks for different T2D datasets		
Dataset	**# of nodes**	**# of edges**
T2D1	119	1356
T2D2	134	1383
T2D3	135	1441
T2DC	158	1709

**Supplementary Table 4:** Identified pathway clusters that are affected in T2D for each dataset.

T2D1			T2D2			T2D3			T2DC		
# of clusters	# of nodes	Score of cluster	# of clusters	# of nodes	Score of cluster	# of clusters	# of nodes	Score of cluster	# of clusters	# of nodes	Score of cluster
7	38	32.919	9	34	30.182	7	35	31.412	8	35	31.118
	14	8.462		19	13.111		21	14.3		16	8.8
	9	4.75		15	5.286		11	5.2		16	8.533
	4	3.333		5	5		5	4.5		11	5
	3	3		5	4,5		4	4		5	5
	3	3		4	4		4	3.333		8	4.286
	3	3		3	3		3	3		4	3.333

**Supplementary Table 5:** The Kappa scores and the numbers of common genes between each pair of consensus T2D pathways.

KEGG ID	KEGG name	KEGG ID	KEGG name	Kappa score	Common gene number
05214	Glioma	05223	Nonsmall cell lung cancer	0.674842	41
05212	Pancreatic cancer	05220	Chronic myeloid leukemia	0.585755	41
05214	Glioma	05220	Chronic myeloid leukemia	0.560827	39
05214	Glioma	05215	Prostate cancer	0.553567	43
04012	ErbB signaling pathway	05214	Glioma	0.547740	42
05212	Pancreatic cancer	05223	Nonsmall cell lung cancer	0.536905	33
05220	Chronic myeloid leukemia	05221	Acute myeloid leukemia	0.534126	35
05220	Chronic myeloid leukemia	05223	Nonsmall cell lung cancer	0.522735	34
05215	Prostate cancer	05223	Nonsmall cell lung cancer	0.505403	37
04012	ErbB signaling pathway	05220	Chronic myeloid leukemia	0.494162	40
05215	Prostate cancer	05221	Acute myeloid leukemia	0.487980	36
05215	Prostate cancer	05220	Chronic myeloid leukemia	0.487857	40
04012	ErbB signaling pathway	05223	Nonsmall cell lung cancer	0.470279	34
05221	Acute myeloid leukemia	05223	Nonsmall cell lung cancer	0.455708	26
05212	Pancreatic cancer	05214	Glioma	0.452808	30
05212	Pancreatic cancer	05215	Prostate cancer	0.445505	35
04012	ErbB signaling pathway	05221	Acute myeloid leukemia	0.438825	32
04722	Neurotrophin signaling pathway	05214	Glioma	0.425391	40
05214	Glioma	05221	Acute myeloid leukemia	0.421118	26
05212	Pancreatic cancer	05221	Acute myeloid leukemia	0.417586	26
04012	ErbB signaling pathway	04722	Neurotrophin signaling pathway	0.416569	44
04722	Neurotrophin signaling pathway	05220	Chronic myeloid leukemia	0.375176	37
00071	Fatty acid degradation	00280	Valine, leucine and isoleucine degradation	0.369459	17
04012	ErbB signaling pathway	04910	Insulin signaling pathway	0.369013	43
04012	ErbB signaling pathway	05215	Prostate cancer	0.366904	33
04722	Neurotrophin signaling pathway	04910	Insulin signaling pathway	0.333639	45
04910	Insulin signaling pathway	04930	Type II diabetes mellitus	0.333501	32
04910	Insulin signaling pathway	05214	Glioma	0.332856	35
04012	ErbB signaling pathway	05212	Pancreatic cancer	0.332587	26
04722	Neurotrophin signaling pathway	05223	Nonsmall cell lung cancer	0.322021	29
04910	Insulin signaling pathway	05220	Chronic myeloid leukemia	0.319142	35
04012	ErbB signaling pathway	04510	Focal adhesion	0.307397	47
04722	Neurotrophin signaling pathway	05215	Prostate cancer	0.305474	33
04722	Neurotrophin signaling pathway	05221	Acute myeloid leukemia	0.297190	27
04910	Insulin signaling pathway	05221	Acute myeloid leukemia	0.296282	30
00071	Fatty acid degradation	03320	PPAR signaling pathway	0.295383	17
04722	Neurotrophin signaling pathway	05212	Pancreatic cancer	0.281428	27
04510	Focal adhesion	05214	Glioma	0.261438	37
04910	Insulin signaling pathway	05215	Prostate cancer	0.259024	31
04910	Insulin signaling pathway	05223	Nonsmall cell lung cancer	0.256664	26
04062	Chemokine signaling pathway	05220	Chronic myeloid leukemia	0.255785	35
03320	PPAR signaling pathway	04920	Adipocytokine signaling pathway	0.251432	18
04062	Chemokine signaling pathway	04722	Neurotrophin signaling pathway	0.249358	41
04910	Insulin signaling pathway	04920	Adipocytokine signaling pathway	0.246928	27
04510	Focal adhesion	05215	Prostate cancer	0.243062	38
04930	Type II diabetes mellitus	05212	Pancreatic cancer	0.239467	14
04012	ErbB signaling pathway	04062	Chemokine signaling pathway	0.233097	34
04510	Focal adhesion	04722	Neurotrophin signaling pathway	0.227594	40
04062	Chemokine signaling pathway	05221	Acute myeloid leukemia	0.225918	29
04930	Type II diabetes mellitus	05221	Acute myeloid leukemia	0.222682	12
04910	Insulin signaling pathway	05212	Pancreatic cancer	0.213040	23
04510	Focal adhesion	05223	Nonsmall cell lung cancer	0.210418	29
00010	Glycolysis / Gluconeogenesis	00071	Fatty acid degradation	0.210120	12
04110	Cell cycle	05220	Chronic myeloid leukemia	0.202547	21
04062	Chemokine signaling pathway	05214	Glioma	0.201371	27
04510	Focal adhesion	05212	Pancreatic cancer	0.200829	29
04062	Chemokine signaling pathway	05212	Pancreatic cancer	0.200395	27
04012	ErbB signaling pathway	04930	Type II diabetes mellitus	0.200216	14
04062	Chemokine signaling pathway	04510	Focal adhesion	0.199227	44
04920	Adipocytokine signaling pathway	04930	Type II diabetes mellitus	0.196741	12
04062	Chemokine signaling pathway	05215	Prostate cancer	0.194466	29
04062	Chemokine signaling pathway	04910	Insulin signaling pathway	0.193923	35
04510	Focal adhesion	04910	Insulin signaling pathway	0.193683	37
04630	JAK-STAT signaling pathway	05220	Chronic myeloid leukemia	0.189152	23
04930	Type II diabetes mellitus	05214	Glioma	0.188174	11
04510	Focal adhesion	05220	Chronic myeloid leukemia	0.187250	28
04930	Type II diabetes mellitus	05223	Nonsmall cell lung cancer	0.186194	10
04062	Chemokine signaling pathway	05223	Nonsmall cell lung cancer	0.185694	24
04930	Type II diabetes mellitus	05215	Prostate cancer	0.182369	13
04722	Neurotrophin signaling pathway	04930	Type II diabetes mellitus	0.182330	16
04630	JAK-STAT signaling pathway	05221	Acute myeloid leukemia	0.177817	20
04930	Type II diabetes mellitus	05220	Chronic myeloid leukemia	0.174873	11
00071	Fatty acid degradation	04920	Adipocytokine signaling pathway	0.168912	10
04920	Adipocytokine signaling pathway	05212	Pancreatic cancer	0.168257	12
04510	Focal adhesion	05221	Acute myeloid leukemia	0.155698	22
04110	Cell cycle	04350	TGF-beta signaling pathway	0.154720	17
04012	ErbB signaling pathway	04630	JAK-STAT signaling pathway	0.150823	20
04920	Adipocytokine signaling pathway	05221	Acute myeloid leukemia	0.149712	10
04110	Cell cycle	05212	Pancreatic cancer	0.147216	15
04722	Neurotrophin signaling pathway	04920	Adipocytokine signaling pathway	0.136266	14
04020	Calcium signaling pathway	05214	Glioma	0.134931	18
04630	JAK-STAT signaling pathway	05212	Pancreatic cancer	0.132448	16
04630	JAK-STAT signaling pathway	05223	Nonsmall cell lung cancer	0.131103	15
04920	Adipocytokine signaling pathway	05220	Chronic myeloid leukemia	0.130829	10
04350	TGF-beta signaling pathway	05212	Pancreatic cancer	0.127816	10
04110	Cell cycle	05215	Prostate cancer	0.127707	15
04630	JAK-STAT signaling pathway	05215	Prostate cancer	0.124352	17
04630	JAK-STAT signaling pathway	05214	Glioma	0.124065	15
04630	JAK-STAT signaling pathway	04910	Insulin signaling pathway	0.123086	21
04350	TGF-beta signaling pathway	05220	Chronic myeloid leukemia	0.120966	10
04920	Adipocytokine signaling pathway	05215	Prostate cancer	0.115713	10
00010	Glycolysis / Gluconeogenesis	04930	Type II diabetes mellitus	0.114537	7
00010	Glycolysis / Gluconeogenesis	04910	Insulin signaling pathway	0.113934	13
04630	JAK-STAT signaling pathway	04930	Type II diabetes mellitus	0.108132	12
04110	Cell cycle	05214	Glioma	0.105312	11
04020	Calcium signaling pathway	04310	Wnt signaling pathway	0.104504	20
04012	ErbB signaling pathway	04310	Wnt signaling pathway	0.100507	13
04062	Chemokine signaling pathway	04930	Type II diabetes mellitus	0.099688	13
00010	Glycolysis / Gluconeogenesis	00280	Valine, leucine and isoleucine degradation	0.098022	6
04062	Chemokine signaling pathway	04630	JAK-STAT signaling pathway	0.093423	20
04510	Focal adhesion	04930	Type II diabetes mellitus	0.091674	13
04630	JAK-STAT signaling pathway	04722	Neurotrophin signaling pathway	0.090770	15
04110	Cell cycle	05223	Nonsmall cell lung cancer	0.089793	9
04920	Adipocytokine signaling pathway	05223	Nonsmall cell lung cancer	0.086981	6
04310	Wnt signaling pathway	05212	Pancreatic cancer	0.085159	10
04310	Wnt signaling pathway	04722	Neurotrophin signaling pathway	0.082773	13
04012	ErbB signaling pathway	04020	Calcium signaling pathway	0.081722	13
04110	Cell cycle	04310	Wnt signaling pathway	0.080919	13
04062	Chemokine signaling pathway	04920	Adipocytokine signaling pathway	0.078990	12
04012	ErbB signaling pathway	04920	Adipocytokine signaling pathway	0.078784	7
04310	Wnt signaling pathway	04350	TGF-beta signaling pathway	0.077256	10
04310	Wnt signaling pathway	05214	Glioma	0.075883	9
04310	Wnt signaling pathway	04510	Focal adhesion	0.075541	17
04310	Wnt signaling pathway	04330	Notch signaling pathway	0.075502	8
04310	Wnt signaling pathway	05215	Prostate cancer	0.072669	10
04020	Calcium signaling pathway	04910	Insulin signaling pathway	0.072522	15
04510	Focal adhesion	04630	JAK-STAT signaling pathway	0.069606	17
04020	Calcium signaling pathway	04722	Neurotrophin signaling pathway	0.067145	13
04020	Calcium signaling pathway	04062	Chemokine signaling pathway	0.067143	17
04630	JAK-STAT signaling pathway	04920	Adipocytokine signaling pathway	0.066537	9
04350	TGF-beta signaling pathway	05221	Acute myeloid leukemia	0.063936	5
00010	Glycolysis / Gluconeogenesis	04920	Adipocytokine signaling pathway	0.063675	5
04062	Chemokine signaling pathway	04310	Wnt signaling pathway	0.063207	14
00280	Valine, leucine and isoleucine degradation	03320	PPAR signaling pathway	0.061337	4
04310	Wnt signaling pathway	05221	Acute myeloid leukemia	0.059998	7
04330	Notch signaling pathway	05220	Chronic myeloid leukemia	0.058187	4
03320	PPAR signaling pathway	05223	Nonsmall cell lung cancer	0.055513	4
04310	Wnt signaling pathway	05220	Chronic myeloid leukemia	0.052512	7
04920	Adipocytokine signaling pathway	05214	Glioma	0.049949	4
04012	ErbB signaling pathway	04350	TGF-beta signaling pathway	0.048351	5
04020	Calcium signaling pathway	05223	Nonsmall cell lung cancer	0.047497	7
04020	Calcium signaling pathway	04930	Type II diabetes mellitus	0.042080	6
04310	Wnt signaling pathway	05223	Nonsmall cell lung cancer	0.039858	5
04350	TGF-beta signaling pathway	04930	Type II diabetes mellitus	0.038491	3
04310	Wnt signaling pathway	04910	Insulin signaling pathway	0.037561	8
04110	Cell cycle	04330	Notch signaling pathway	0.036823	4
04350	TGF-beta signaling pathway	05215	Prostate cancer	0.035527	4
04012	ErbB signaling pathway	04110	Cell cycle	0.033022	5
04020	Calcium signaling pathway	04510	Focal adhesion	0.029693	11
04510	Focal adhesion	04920	Adipocytokine signaling pathway	0.028550	6
03320	PPAR signaling pathway	04910	Insulin signaling pathway	0.025182	4
04110	Cell cycle	04630	JAK-STAT signaling pathway	0.023244	6
04330	Notch signaling pathway	04350	TGF-beta signaling pathway	0.022730	2
04110	Cell cycle	05221	Acute myeloid leukemia	0.022049	3
04350	TGF-beta signaling pathway	04910	Insulin signaling pathway	0.021892	4
04310	Wnt signaling pathway	04930	Type II diabetes mellitus	0.021752	3
04330	Notch signaling pathway	05215	Prostate cancer	0.020317	2
04350	TGF-beta signaling pathway	05223	Nonsmall cell lung cancer	0.020029	2
00010	Glycolysis / Gluconeogenesis	03320	PPAR signaling pathway	0.019725	2
04310	Wnt signaling pathway	04630	JAK-STAT signaling pathway	0.019514	6
04350	TGF-beta signaling pathway	04630	JAK-STAT signaling pathway	0.018819	4
04350	TGF-beta signaling pathway	04510	Focal adhesion	0.018382	5
04350	TGF-beta signaling pathway	05214	Glioma	0.017344	2
04350	TGF-beta signaling pathway	04722	Neurotrophin signaling pathway	0.016276	3
04110	Cell cycle	04722	Neurotrophin signaling pathway	0.015333	4
04310	Wnt signaling pathway	04920	Adipocytokine signaling pathway	0.015212	3

**Supplementary Table 6:** Possible T2D causing genes that are both highly represented in our analysis within the gold-standard pathways of T2D and also found in DisGeNET as associated with T2D.

ABCC8	ADRB3	CACNA1D	EPO	HNF4A	IRS2	NPY	PPARA	SLC2A4
ACACA	AGTR1	CAMKK2	ERBB4	HSD17B12	KCNJ11	NR1D1	PPARD	SOCS3
ACACB	AKT1	CCND2	FADS2	IL10	LEPR	NR1H3	PPARG	SORBS1
ACSL1	AKT2	CDKN2A	FASN	IL23R	LPL	ONECUT1	PPARGC1A	SREBF1
ADCY3	AKT3	CDKN2B	FOXO1	IL4R	MCM6	PCK1	PPP1R3A	TACR3
ADIPOQ	ALDH2	CHEK2	GCK	IL6	NEUROD1	PDX1	PRKAA2	TCF7L2
ADIPOR1	ALDH7A1	CHRM3	GYS1	IL6R	NEUROG3	PIK3CB	PTPN1	TGFB1
ADIPOR2	ARNTL	CLOCK	HK2	INS	NFKB1	PKLR	RAPGEF1	TNF
ADRA1A	BCAT1	CRY2	HNF1A	INSR	NOS3	PLIN1	SLC2A1	TP53
ADRB2	BRAF	CTBP1	HNF1B	IRS1	NOTCH2	PLTP	SLC2A2	WNT5B

**Supplementary Table 7:** Potential T2D causing genes that are highly represented in the gold-standard pathways of T2D in our analy in our analysis.

	Frequencies in the gold-standard pathways of T2D, obtained using different datasets					Glycolysis / Gluconeogenesis	Fatty acid metabolism	Valine, leucine and isoleucine degradation	Valine, leucine and isoleucine biosynthesis	Biosynthesis of unsaturated fatty acids	PPAR signaling pathway	Calcium signaling pathway	Cell cycle	Wnt signaling pathway	Notch signaling pathway	JAK-STAT signaling pathway
Gene	Total	T2D1	T2D2	T2D3	T2DC	hsa00010	hsa00071	hsa00280	hsa00290	hsa01040	hsa03320	hsa04020	hsa04110	hsa04310	hsa04330	hsa04630
ACAA1	14	4	2	4	4		✓	✓			✓					
ACADM	11	3	2	3	3		✓	✓			✓					
ACOX1	12	3	3	3	3		✓				✓					
ACSL1	12	3	3	3	3		✓				✓					
ACSL3	12	3	3	3	3		✓				✓					
ACSL5	11	3	3	3	2		✓				✓					
AKT2	12	3	3	3	3											✓
AKT3	12	3	3	3	3											✓
ALDH1B1	12	3	3	3	3	✓	✓	✓								
ALDH2	12	3	3	3	3	✓	✓	✓								
ALDH3A2	12	3	3	3	3	✓	✓	✓								
ALDH7A1	12	3	3	3	3	✓	✓	✓								
ALDH9A1	12	3	3	3	3	✓	✓	✓								
CPT1A	12	3	3	3	3		✓				✓					
CPT1B	12	3	3	3	3		✓				✓					
CREBBP	15	4	4	3	4								✓	✓	✓	✓
EHHADH	11	3	2	3	3		✓	✓			✓					
EP300	14	4	3	3	4								✓	✓	✓	✓
GCK	12	3	3	3	3	✓										
GSK3B	12	3	3	3	3								✓	✓		
IKBKB	12	3	3	3	3											
IRS1	12	3	3	3	3											
IRS2	12	3	3	3	3											
MAPK10	15	4	4	3	4									✓		
MAPK8	15	4	4	3	4									✓		
MAPK9	15	4	4	3	4									✓		
MYC	12	3	3	3	3								✓	✓		✓
PIK3CA	12	3	3	3	3											✓
PIK3CB	12	3	3	3	3											✓
PIK3CD	12	3	3	3	3											✓
PIK3R1	12	3	3	3	3											✓
PIK3R2	12	3	3	3	3											✓
PIK3R3	12	3	3	3	3											✓

## Figures and Tables

**Table 1:** Contingency table of overlapping genes (n_i,j_) between subnetworks U_i_ and V_j_, where U and V indicate the sets of subnetworks identified via using datasets X and Y, respectively.

U | V	V_1_ V_2_ … V_S_	Sum
U_1_	n_11_ n_12_ … n_1S_	a_1_
U_2_	n_21_ n_22_ … n_2S_	a_2_
…	… … … …	…
U_R_	n_R1_ n_R2_ … n_RS_	a_R_
Sum	b_1_ b_2_ … b_S_	N

**Table 2:** Summary of T2D1, T2D2, T2D3, T2DC datasets, and the numbers of identified SNPs, genes, subnetworks for each dataset.

Datasets	# of cases	# of controls	# of SNPs	# of SNPs (p-value < 0.05)	# of rsIDs	# of genes	# of subnetworks
**T2D1**	12.931	57.196	14.683.492	762.111	335.212	15.806	984
**T2D2**	62.892	596.424	5.053.015	557.564	557.564	15.460	904
**T2D3**	74.124	824.006	21.635.866	1.525.650	639.622	17.800	941
**T2DC**	-	-	-	-	-	10.298	813

**Table 3:** Top 10 affected T2D pathways of T2DC dataset. Among these pathways, 5 pathways (shown in bold) are commonly overrepresented for the dysregulated modules of T2D1, T2D2, T2D3, T2DC datasets.

	Rank				# of genes identified in different datasets (DD)				# of genes found in at least one dataset (Union)	# of genes in pathway (GiP)	Percent of identified genes in pathways	
KEGG term	T2DC	T2D1	T2D2	T2D3	T2DC	T2D1	T2D2	T2D3			max (DD) / GiP	Union / GiP
**Spliceosome**	1	15	8	5	65	62	75	**85**	104	127	0.66	0.81
**Focal adhesion**	2	10	1	1	**150**	135	146	146	172	200	0.75	0.86
**SNARE interactions in vesicular transport**	3	3	5	4	31	30	29	**32**	34	36	0.88	0.94
**Valine leucine and isoleucine degradation**	4	1	34	13	36	36	35	**37**	41	44	0.84	0.93
**Purine metabolism**	5	2	83	3	54	57	34	**92**	99	166	0.55	0.59
**Dopaminergic synapse**	6	37	7	9	107	107	**110**	103	119	130	0.84	0.91
**TGF-beta signaling pathway**	7	6	3	15	62	**64**	**64**	58	75	84	0.76	0.89
**ErbB signaling pathway**	8	8	9	7	**84**	80	82	81	85	87	0.96	0.97
**Chemokine signaling pathway**	9	33	20	39	107	**139**	111	129	163	189	0.73	0.86
**Glutamatergic synapse**	10	38	10	14	81	86	**88**	87	101	126	0.69	0.80

**Table 4:** Comparison of the overrepresented pathways of T2D dysregulated modules (T2DC), expanded modules of T2D seed genes (T2D_D500), the affected pathways identified using Pascal (T2DP). While the pathways, which are highlighted in bold refers to the gold standard T2D KEGG pathways reported in Yoon et al.’s (2018) study; the pathways, which are highlighted in italic refers to the pathways that are not included in gold standard T2D KEGG pathways, but they have support from literature as related with T2D.

	p-value			Rank		
KEGG term	T2DP	T2DC	T2D_D500	T2DP	T2DC	T2D_D500
**Pathways in cancer**	1.42E-15	2.52E-20	1.86E-33	2	24	79
**Focal adhesion**	4.39E-14	7.03E-38	1.48E-33	3	2	80
**Type II diabetes mellitus**	4.72E-14	1.84E-08	1.81E-10	4	127	43
**Prostate cancer**	4.28E-10	1.19E-19	2.94E-29	7	27	73
**Calcium signaling pathway**	9.66E-10	3.71E-13	2.18E-08	9	77	33
**MAPK signaling pathway**	3.48E-08	8.59E-24	5.25E-27	10	14	71
**Small cell lung cancer**	7.44E-08	5.10E-10	1.79E-07	11	110	26
**Chronic myeloid leukemia**	7.78E-08	5.65E-19	1.09E-31	12	33	77
**Insulin signaling pathway**	2.12E-07	2.67E-14	2.21E-30	13	63	76
**Glioma**	3.01E-07	7.22E-18	6.81E-32	14	36	78
**Nonsmall cell lung cancer**	7.16E-07	6.51E-12	3.38E-26	15	87	70
***GnRH signaling pathway***	1.93E-06	1.81E-19	8.73E-20	17	29	62
***Pancreatic cancer***	2.41E-06	4.22E-15	4.55E-21	18	56	65
**Vascular smooth muscle contraction**	2.80E-06	1.21E-19	1.41E-05	19	28	19
**Leukocyte transendothelial migration**	6.45E-06	2.82E-13	2.35E-16	20	76	53
**Chemokine signaling pathway**	8.94E-06	5.24E-28	1.70E-29	21	9	74
**Gap junction**	3.33E-05	1.17E-20	5.05E-08	23	23	31
**Tight junction**	9.78E-05	6.68E-14	1.35E-09	25	67	39
**Wnt signaling pathway**	1.16E-04	5.63E-22	3.97E-06	26	21	22
**Adipocytokine signaling pathway**	1.35E-04	5.40E-11	1.35E-05	27	95	20
**Acute myeloid leukemia**	1.55E-04	1.08E-13	4.62E-21	29	72	63
***Adherens junction***	1.61E-04	2.81E-24	7.02E-24	30	12	67
**Long-term depression**	2.23E-04	1.67E-16	2.98E-06	31	46	62
***ErbB signaling pathway***	2.81E-04	1.60E-28	2.74E-54	32	8	83
***Phosphatidylinositol signaling system***	3.49E-04	1.91E-23	1.05E-02	33	16	2
***Neurotrophin signaling pathway***	3.91E-04	3.03E-22	2.08E-58	34	20	84
**Melanogenesis**	4.38E-04	1.81E-19	1.57E-07	36	30	27
**JAK-STAT signaling pathway**	4.57E-04	7.54E-14	6.66E-19	37	68	60
**Long-term potentiation**	6.07E-04	3.64E-15	9.56E-19	38	55	26

**Table 5:** Common pathways of highest scoring pathway clusters identified for different T2D GWAS metadata.

Pathway name	p-values				Rank			
	T2D1	T2D2	T2D3	T2DC	T2D1	T2D2	T2D3	T2DC
**Renal cell carcinoma**	7.12E-15	1.95E-15	7.23E-13	8.14E-15	68	55	90	57
**Colorectal cancer**	1.52E-12	7.53E-10	1.82E-14	3.51E-17	97	115	77	41
**Hepatitis C**	2.99E-14	1.29E-14	1.35E-18	1.59E-16	77	62	47	43
**VEGF signaling pathway**	1.05E-11	1.20E-10	4.18E-12	4.15E-13	104	99	99	78
**Toxoplasmosis**	2.38E-12	2.24E-12	1.30E-18	4.39E-13	99	78	48	80
**Chagas disease (American trypanosomiasis)**	2.10E-18	1.62E-12	3.85E-19	3.57E-15	48	76	42	54
**Type II diabetes mellitus**	1.32E-12	2.68E-09	6.18E-19	1.84E-08	96	124	44	127
**Chemokine signaling pathway**	1.47E-21	1.01E-23	2.97E-19	5.23E-28	33	20	39	9
**Progesterone-mediated oocyte maturation**	2.67E-16	3.57E-12	4.95E-16	7.25E-18	62	81	68	37
**Insulin signaling pathway**	2.16E-16	1.67E-16	2.96E-18	2.67E-14	60	48	49	63
**Toll-like receptor signaling pathway**	1.70E-29	2.63E-11	3.20E-13	1.27E-14	13	91	85	62
**Cholinergic synapse**	6.32E-35	1.17E-25	1.61E-31	4.37E-27	4	16	11	11
**Neurotrophin signaling pathway**	4.20E-22	3.68E-23	3.03E-31	3.02E-22	30	22	12	20
**Fc gamma R-mediated phagocytosis**	3.57E-19	2.88E-18	1.01E-19	1.75E-16	44	37	35	47
**Osteoclast differentiation**	5.24E-22	1.28E-14	3.60E-19	3.16E-17	31	61	41	40
**T cell receptor signaling pathway**	3.32E-19	3.69E-21	4.49E-20	2.14E-18	43	32	33	34
**Fc epsilon RI signaling pathway**	3.75E-18	9.42E-16	5.92E-18	2.33E-23	52	53	52	17
**Natural killer cell mediated cytotoxicity**	2.61E-13	1.53E-13	2.12E-09	5.47E-12	90	69	131	86
**B cell receptor signaling pathway**	3.28E-19	3.39E-17	2.41E-14	1.96E-19	42	43	78	31
**mTOR signaling pathway**	1.28E-12	4.34E-10	1.72E-08	1.60E-10	95	108	141	102
**Nonsmall cell lung cancer**	7.60E-16	3.04E-11	1.86E-13	6.51E-12	65	92	82	87
**ErbB signaling pathway**	4.64E-31	1.09E-29	1.46E-37	1.59E-28	8	9	7	8
**Acute myeloid leukemia**	5.42E-14	1.40E-10	1.03E-11	1.08E-13	80	102	105	72
**Chronic myeloid leukemia**	7.27E-20	8.58E-17	2.48E-16	5.65E-19	41	45	65	33
**Melanoma**	4.79E-14	8.51E-17	6.46E-15	1.05E-14	78	44	74	59
**Prostate cancer**	1.13E-17	1.82E-13	1.12E-12	1.18E-19	53	70	93	27
**Glioma**	3.33E-21	1.67E-16	7.34E-19	7.21E-18	35	47	45	36
**Endometrial cancer**	3.47E-16	1.67E-14	4.80E-13	1.62E-16	63	63	88	45
**Pancreatic cancer**	6.15E-13	4.15E-14	8.21E-15	4.21E-15	94	65	75	56

**Table 6:** Consensus list of T2D pathways that are identified using different T2D GWAS metadata and different network and pathway oriented post-GWAS analyze.

KEGG ID	Pathway name
**KEGG:04920**	Adipocytokine signaling pathway
**KEGG:04020**	Calcium signaling pathway
**KEGG:04110**	Cell cycle
**KEGG:04062**	Chemokine signaling pathway
**KEGG:00071**	Fatty acid metabolism
**KEGG:04510**	Focal adhesion
**KEGG:00010**	Glycolysis/gluconeogenesis
**KEGG:04910**	Insulin signaling pathway
**KEGG:04630**	JAK-STAT signaling pathway
**KEGG:04722**	Neurotrophin signaling pathway
**KEGG:04330**	Notch signaling pathway
**KEGG:03320**	PPAR signaling pathway
**KEGG:04130**	SNARE interactions in vesicular transport
**KEGG:03040**	Spliceosome
**KEGG:04350**	TGF-beta signaling pathway
**KEGG:04930**	Type II diabetes mellitus
**KEGG:00280**	Valine, leucine and isoleucine degradation
**KEGG:04310**	Wnt signaling pathway
	Cancer related pathways*

Cancer related pathways*: acute myeloid leukemia, chronic myeloid leukemia, ErbB signaling pathway, glioma, nonsmall cell lung cancer, pancreatic cancer, prostate cancer.

## Data Availability

The data and the useful scripts are available at: https://github.com/MstafaTmz/T2D.

## Abbreviations

GWAS: Genome-wide association study
HWE: Hardy-Weinberg equilibrium
KEGG: Kyoto Encyclopedia of Genes and Genomes
MCODE: Molecular complex detection
Pascal: Pathway scoring algorithm
PPI: Protein-protein interaction
rsIDs: Reference SNP cluster IDs
SNP: Single nucleotide polymorphism
VEST: The Variant Effect Scoring Tool
