# Supplementary figures and images for: Enlightening the molecular mechanisms of type 2 diabetes with a novel pathway clustering and pathway subnetwork approach
Source: Turk J Biol. 2022 Jul 18;46(4):318–41. doi: 10.55730/1300-0152.2620 (PMC10387888; doi:10.55730/1300-0152.2620)

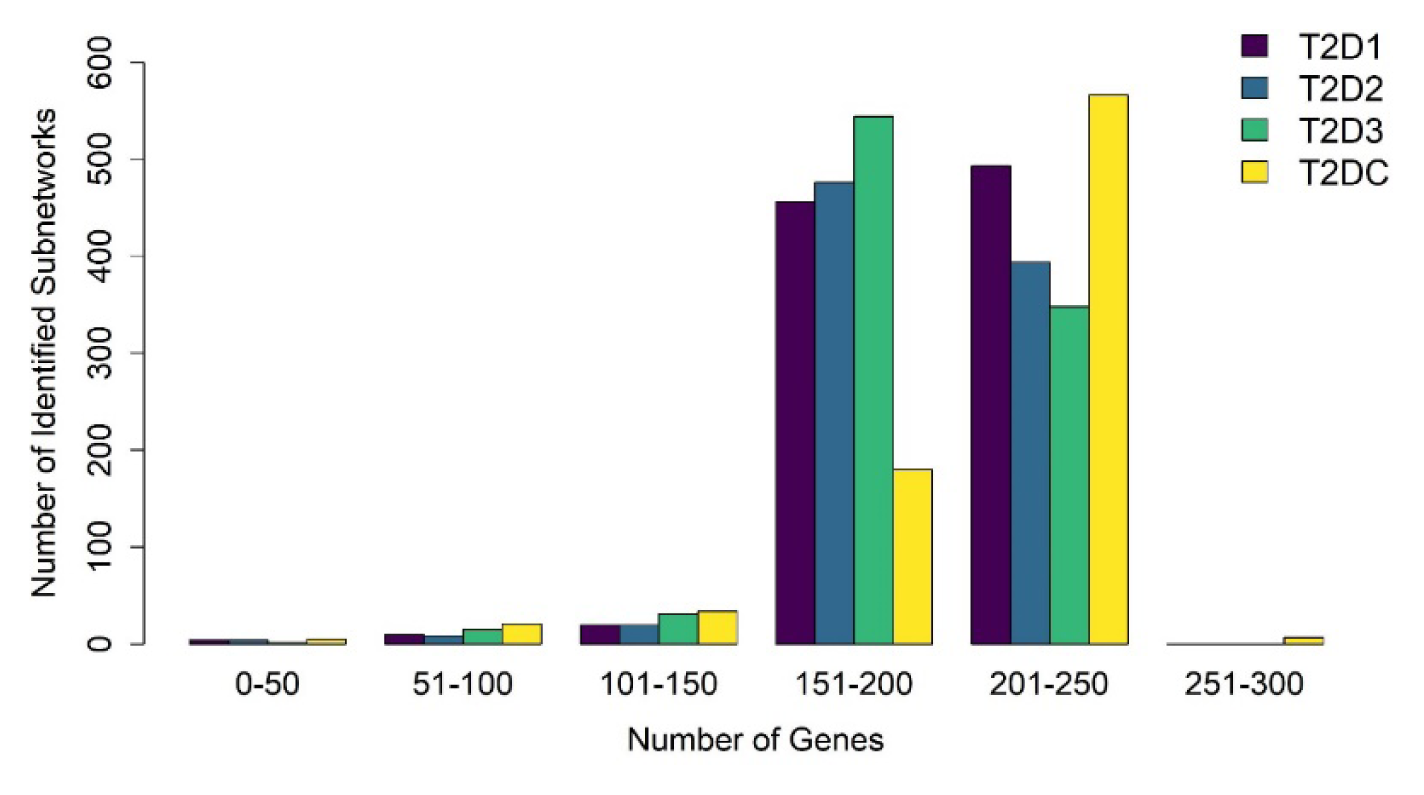

Supplement: Supplemental Figure 1 — Numbers of genes included in the identified (A) 983 subnetworks for T2D1, (B) 903 subnetworks for T2D2, (C) 940 subnetworks for T2D3, and (D) 813 subnetworks for T2DC datasets. [file turkjbiol-46-4-318s1.tif]

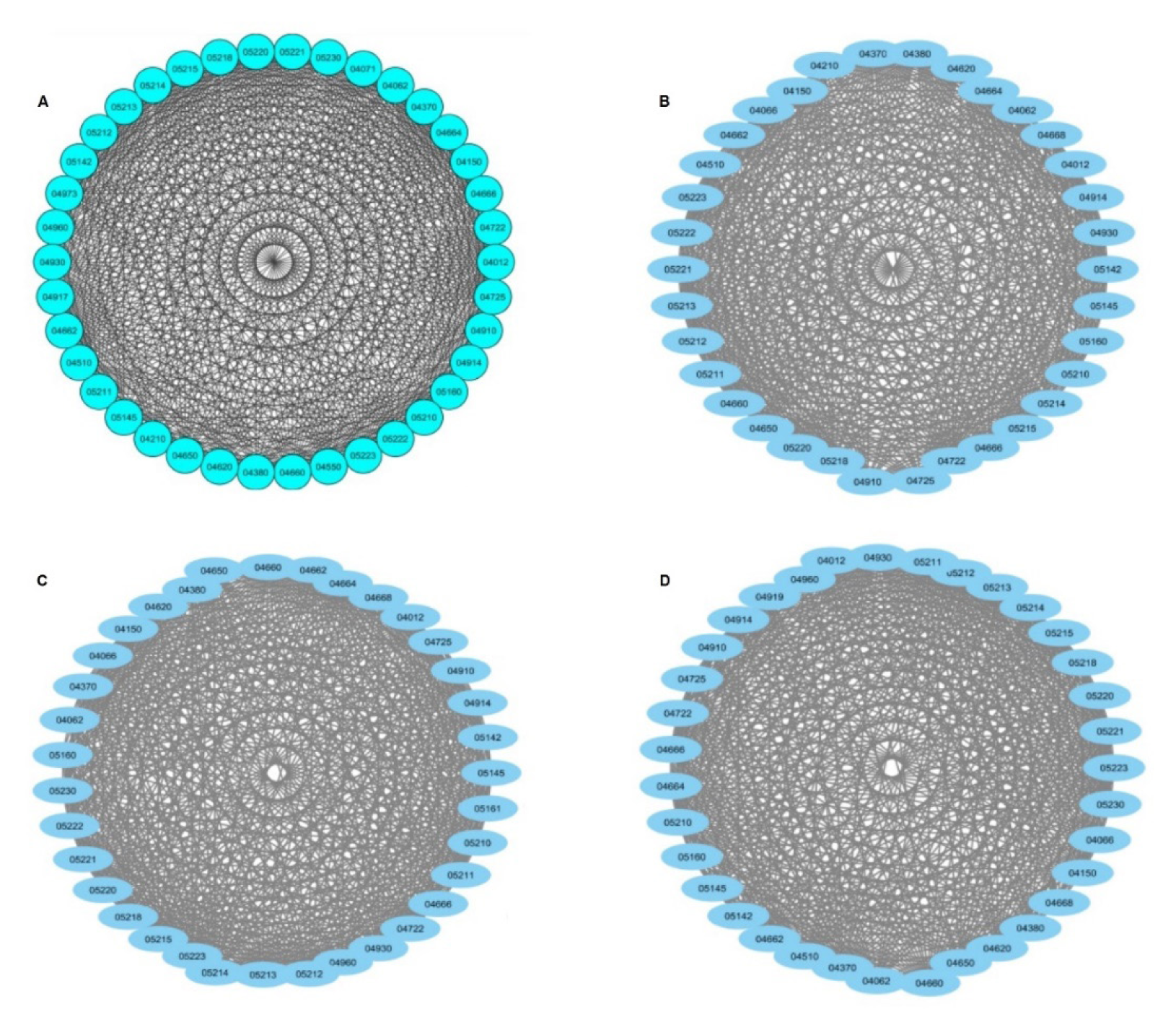

Supplement: Supplemental Figure 2 — The representative networks of the highest scoring pathway clusters of (A) T2D1, (B) T2D2, (C) T2D3, (D) T2DC datasets, including 38, 34, 35 and 35 pathways, respectively. [file turkjbiol-46-4-318s2.tif]

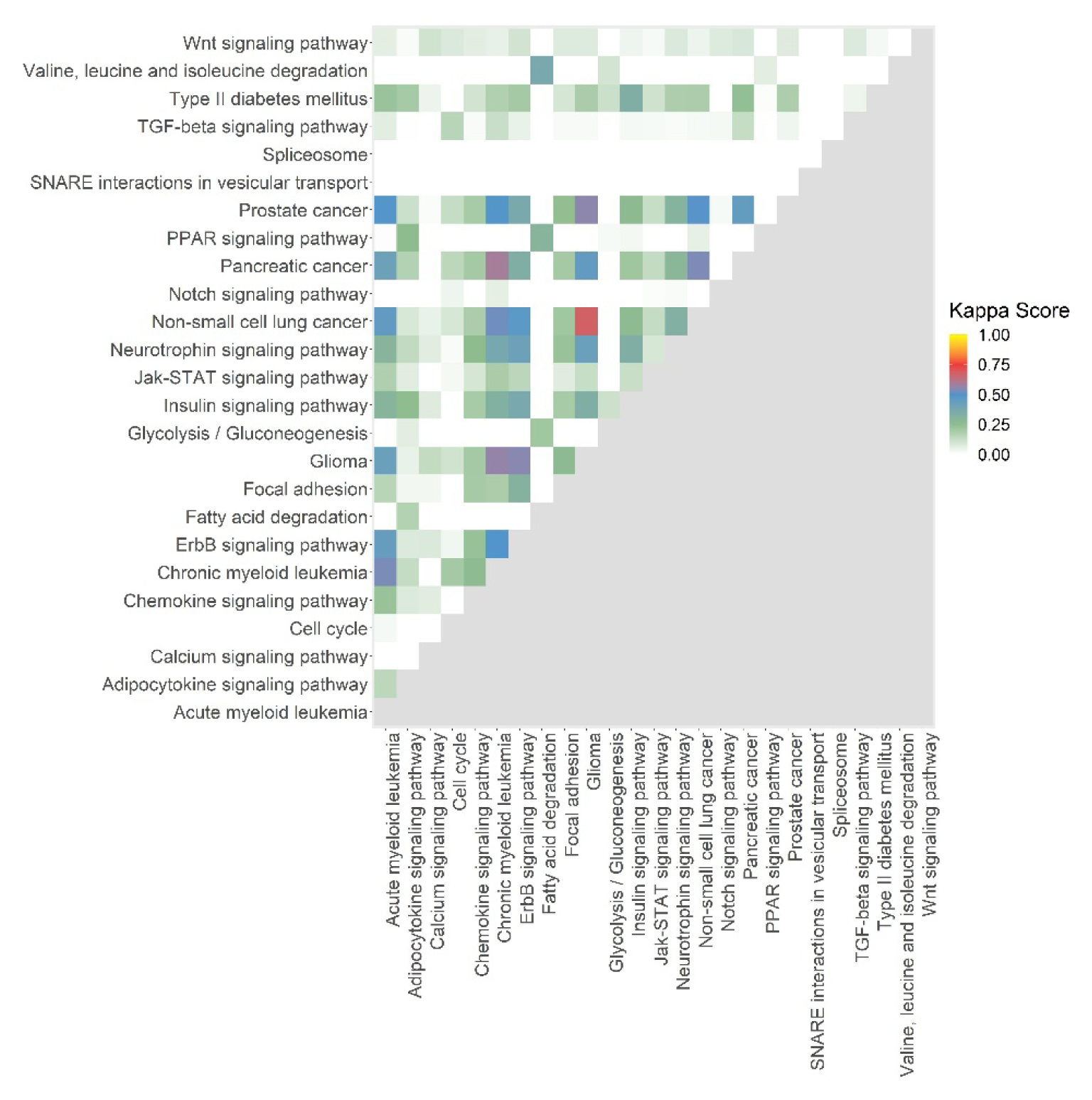

Supplement: Supplemental Figure 3 — The commonalities among our consensus T2D pathways (based on the Kappa scores). While red, purple and blue colors represent higher commonalities between the genes of a pathway pair and also higher Kappa scores; the green color represents less commonality between the genes of a pathway pair and also lower Kappa score for a pathway pair in the heatmap. The white color represents that none of the genes or very small numbers of genes are common between the genes of a pathway pair. [file turkjbiol-46-4-318s3.tif]

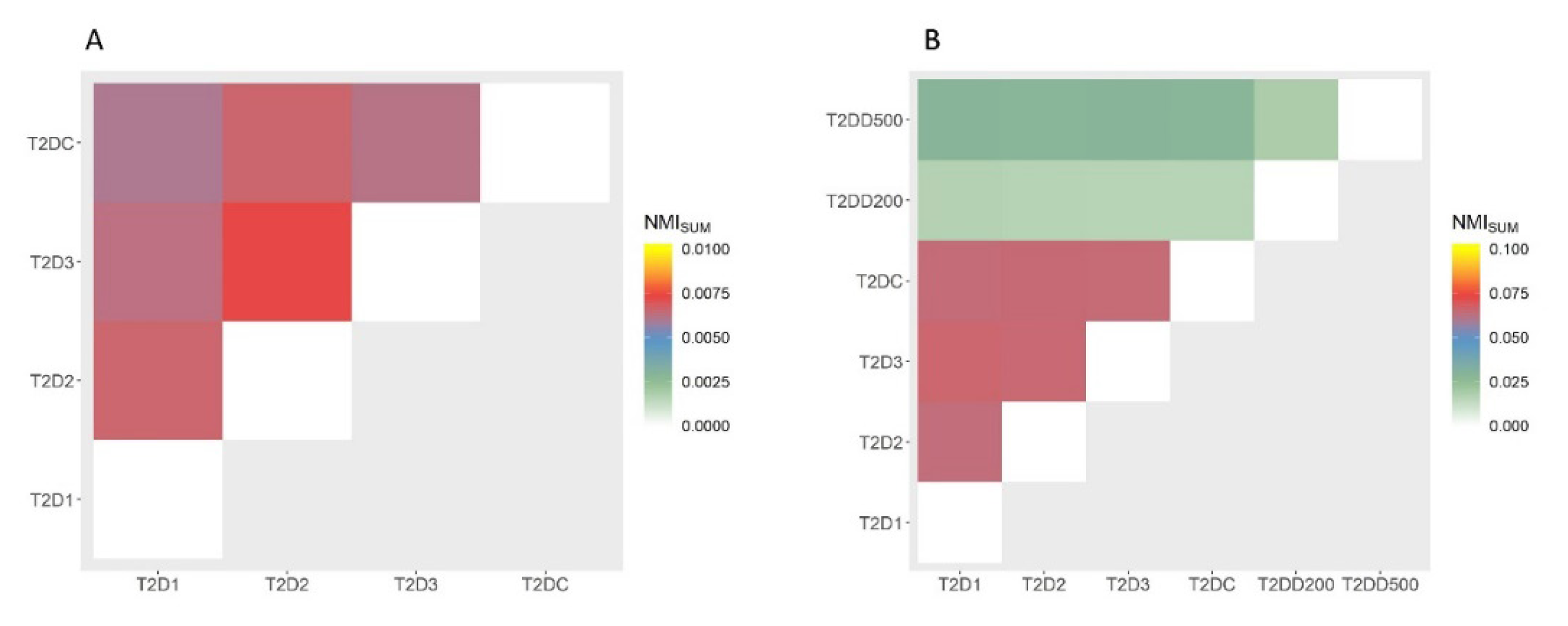

Supplement: Supplemental Figure 4 — Shared information comparison among different datasets in terms of (A) identified T2D subnetworks, and (B) identified pathways via normalized mutual information (NMISUM). While the darker colors indicate higher correlation, lighter colors indicate smaller correlation. NMISUM scores in the diagonals of the heatmap are “whitened” for clearer visibility of the other NMISUM values. [file turkjbiol-46-4-318s4.tif]

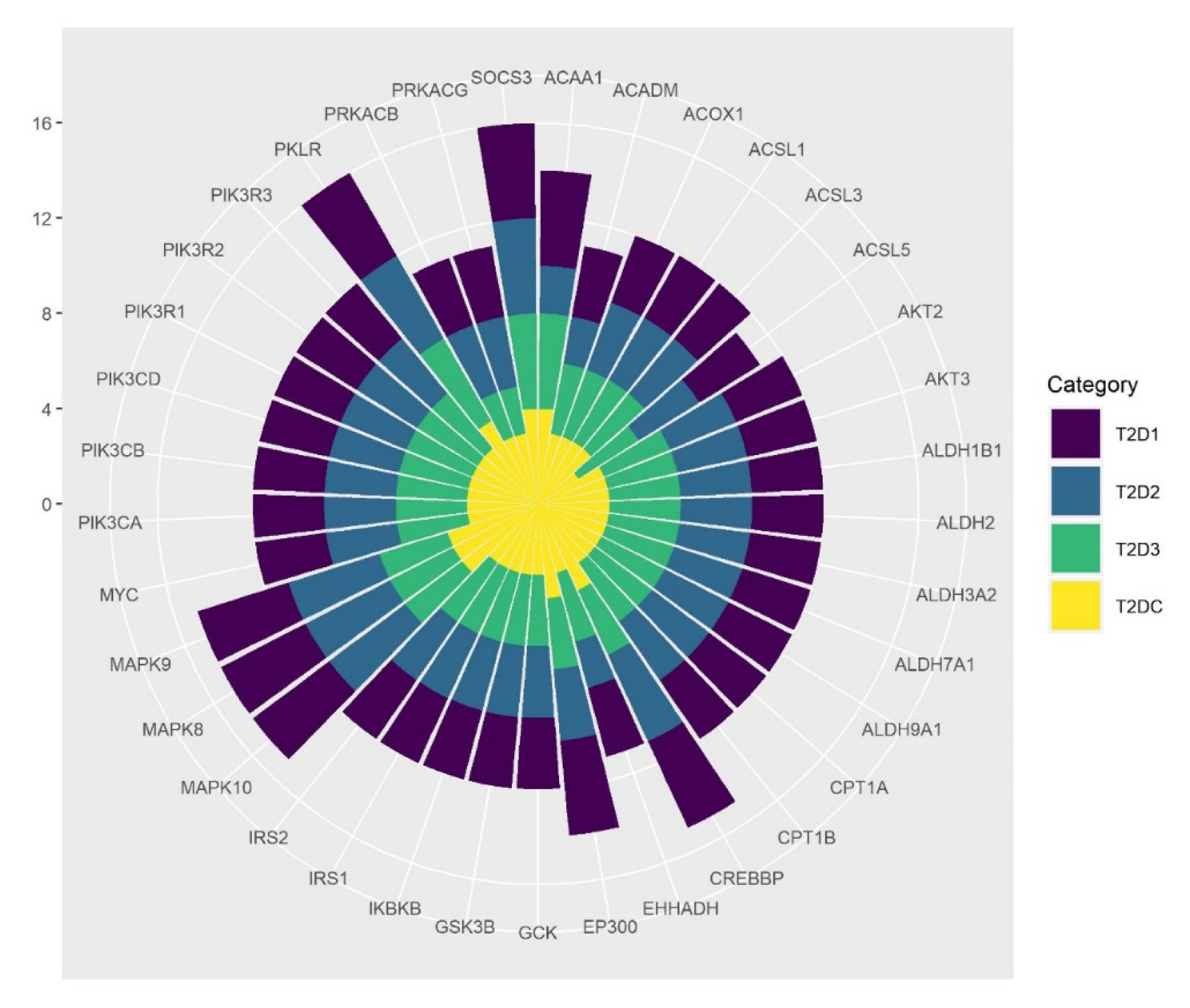

Supplement: Supplemental Figure 5 — Highly targeted T2D genes that reside in gold standard KEGG pathways of T2D. Frequencies in different datasets are shown with different colors. [file turkjbiol-46-4-318s5.tif]
